# Impact of the updated SOFA-2 score on sepsis diagnosis and prognosis: a retrospective multicenter cohort study

**DOI:** 10.1186/s13054-026-06070-1

**Published:** 2026-05-06

**Authors:** Haibo Zhu, Peirong Li, Bing Wang, Hao Fu, Yehan Guo, Ziyi Han, Shuojing Huang, Yujie Xie, Jun He, Shixiang Zheng, Xiaopei Shen

**Affiliations:** 1 Department of Bioinformatics, Fujian Key Laboratory of Medical Bioinformatics, Institute of Precision Medicine, School of Medical Technology and Engineering, Fujian Medical University, University Town Fuzhou, 1st Xueyuan Road, Fuzhou, Fujian 350122 China; 2 The School of Basic Medical Sciences, Fujian Medical University, Fuzhou, China; 3 Key Laboratory of Gastrointestinal Cancer, Fujian Medical University, Ministry of Education, Fuzhou, China; 4 School of Medical Imaging, Fujian Medical University, Fuzhou, China; 5 Department of Emergency Medicine, The Second Affiliated Hospital of Fujian University of Traditional Chinese Medicine, Fuzhou, Fujian 350001 China

**Keywords:** Sepsis, Sequential Organ Failure Assessment, SOFA-2, Sepsis-3, Intensive Care Units, Prognostic Models

## Abstract

**Background:**

The Sepsis-3 criteria operationalized organ dysfunction using the original Sequential Organ Failure Assessment (SOFA-1) score, which was updated to SOFA-2 in October 2025 to align with modern intensive care unit (ICU) practices. However, the impact of adopting SOFA-2 for sepsis detection under the Sepsis-3 criteria has not yet been evaluated.

**Methods:**

We conducted a retrospective multicenter cohort study using three large-scale ICU databases from the United States and the Netherlands. Adult patients with suspected infection within 72 h of ICU admission were included. Sepsis was independently identified according to Sepsis-3 criteria, utilizing either the SOFA-1 or SOFA-2 score. We systematically compared diagnostic concordance, the timeliness of sepsis detection, clinical outcomes and predictive performance of prognostic models between the two scoring systems. The primary outcome was ICU mortality, while secondary outcomes included hospital mortality and 28-day survival.

**Results:**

The study cohort comprised 74,615 adult patients with suspected infection. The diagnostic concordance of sepsis between SOFA-1 and SOFA-2 reached 89.62%. However, SOFA-1 and SOFA-2 uniquely identified an additional 3.54% and 6.84% of patients as having sepsis, respectively. The diagnostic discrepancies were primarily attributable to updates in respiratory and renal scoring criteria. ICU mortality was highest among the Concordant Positive group (15.63%). Notably, both discordant groups exhibited substantial mortality (SOFA-1 Only: 8.31%; SOFA-2 Only: 9.23%), both of which were significantly higher than those of patients not classified as having sepsis by either score (6.71%; *P* = .002 and *P* = 2.87 × 10^–15^). Within the Concordant Positive group, 61.24% of patients were diagnosed simultaneously by both criteria. However, SOFA-2 achieved earlier diagnosis in a greater proportion of cases than SOFA-1 (23.12% vs. 15.64%), despite heterogeneity across different databases. Predictive models derived from the SOFA-2 score demonstrated numerically higher area under the receiver operating characteristic curve (AUROC) values in forecasting ICU mortality than those based on SOFA-1 (0.736 vs. 0.728 in internal cross-validation, *P* = .386; 0.743 vs. 0.720 in external validation, *P* = .375).

**Conclusions:**

SOFA-1 and SOFA-2 showed high concordance in sepsis detection, yet each identified distinct patient subgroups with significant mortality. A transitional strategy utilizing both SOFA-1 and SOFA-2 is advised until updated expert-validated sepsis criteria are established.

**Supplementary Information:**

The online version contains supplementary material available at 10.1186/s13054-026-06070-1.

## Background

Sepsis is a life-threatening organ dysfunction caused by a dysregulated host response to infection and remains a leading cause of global mortality [1, 2]. The Third International Consensus Definitions for Sepsis and Septic Shock (Sepsis-3) operationalize the concept as suspected infection accompanied by an acute increase of at least 2 points in the Sequential Organ Failure Assessment (SOFA) score [3–5]. Developed in 1996, the original SOFA (hereinafter referred to as SOFA-1) was designed to characterize organ failure reflecting the standards of intensive care practice of that time [6]. However, accumulating evidence indicates a discrepancy between SOFA-1 components and organ dysfunction under modern practice [7, 8]. This misalignment may reduce the sensitivity of Sepsis-3 in detecting early or atypical sepsis cases, suggesting that a recalibration of the SOFA-1 criteria is warranted [9].

Consequently, the SOFA-2 score was released in October 2025 to bridge the gap with contemporary clinical practice [10, 11]. Compared with SOFA-1, SOFA-2 introduces updates across all six organ systems, with particularly significant changes to respiratory and renal assessments. Specifically, the respiratory component incorporates SpO_2_/FiO_2_ ratios [12–14] and non-invasive ventilation (NIV), and the renal component adopts weight-standardized urine output thresholds [15–17]. These updates may substantially alter sepsis detection and risk stratification as SOFA-2 is adopted in place of SOFA-1.

However, prior studies have highlighted challenges in operationalizing the original SOFA score for sepsis surveillance [18–23], and the clinical impact of adopting SOFA-2 for sepsis detection and its prognostic validity remain unevaluated. To address this gap, we conducted this multicenter retrospective study leveraging three large-scale ICU databases. Our study aimed to investigate four aspects: (1) the diagnostic concordance between SOFA-1 and SOFA-2; (2) the clinical characteristics of discordant cases detected by only one of the criteria; (3) the potential for SOFA-2 to facilitate earlier diagnosis; and (4) the predictive performance of both criteria regarding patient mortality. Through this analysis, we aimed to provide empirical evidence for benchmarking the transition from SOFA-1 to SOFA-2 in sepsis diagnosis and prognosis.

## Methods

### Study design and data sources

We conducted a multicenter, retrospective cohort study to systematically compare SOFA-1 and SOFA-2 within the Sepsis-3 framework. For each patient, sepsis status was independently adjudicated by applying SOFA-1–based and SOFA-2–based criteria to the same underlying clinical data stream.

The analyses were performed using data from three large, independent ICU databases: MIMIC-IV (v3.1, 2008–2022) from Beth Israel Deaconess Medical Center in the US, which provides comprehensive data from a single academic center [24]; eICU-CRD (v2.0, 2014–2015), which aggregates data from 208 hospitals across the US, offering a broader scope but with more heterogeneous documentation across contributing sites [25]; and AmsterdamUMCdb (v1.0, 2003–2016) from Amsterdam University Medical Centers in the Netherlands, which includes a substantial proportion of cardiothoracic and other planned postoperative surgical admissions [26]. To improve comparability, a standardized data-processing workflow was applied across all three databases, including unified variable extraction, consistent unit conversion, and harmonized handling of missing data. The analysis was restricted to sepsis detection within 72 h of ICU admission (see eMethods for details).

This study followed the Strengthening the Reporting of Observational Studies in Epidemiology (STROBE) reporting guideline [27]. These datasets are publicly available and their use was approved by the respective institutional review boards.

### Participants

**Fig. 1:**
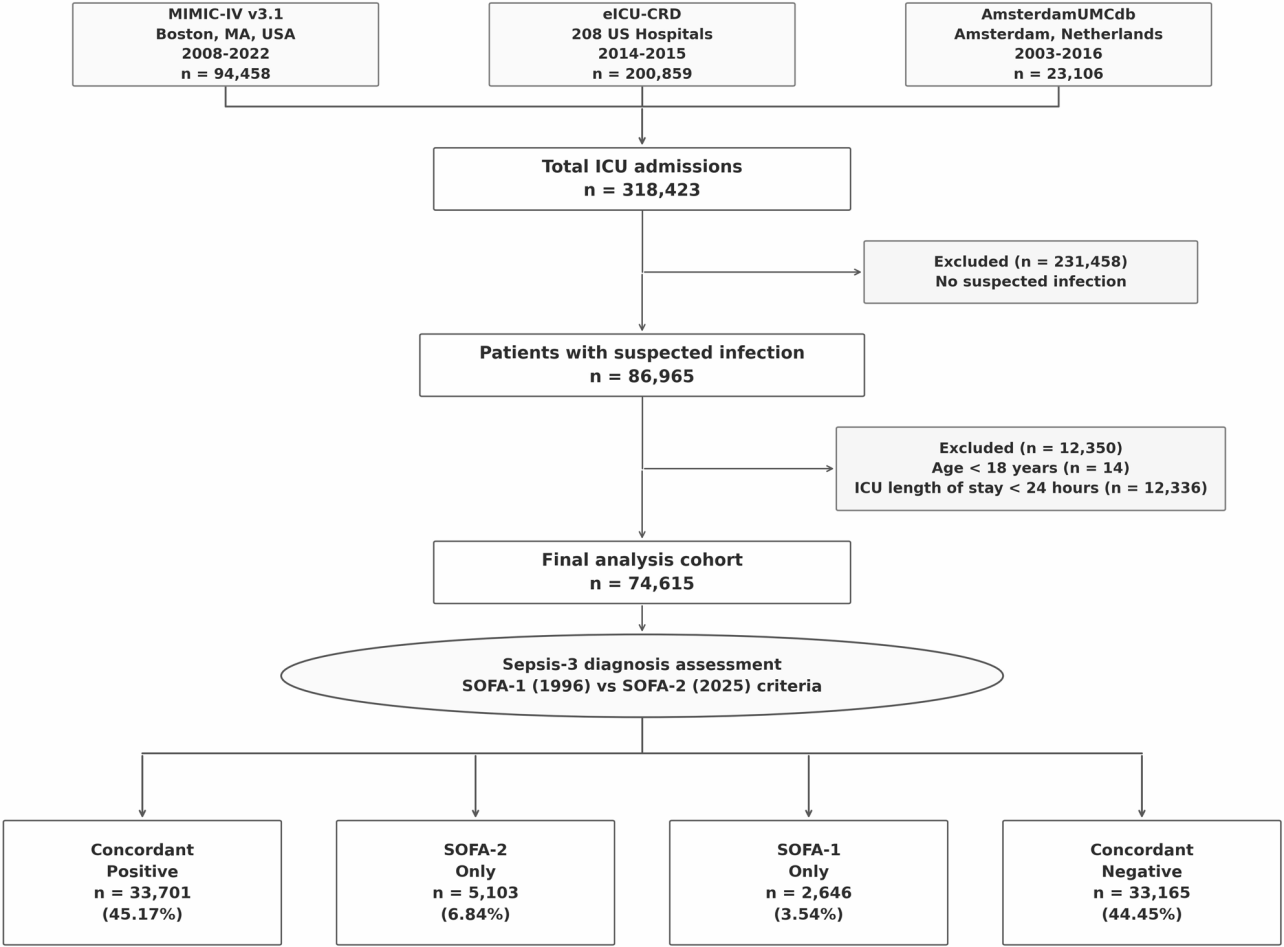
Study flow diagram of cohort selection and patient stratification. Patients were screened from three ICU databases and classified according to sepsis detection by SOFA-1 and SOFA-2 into concordant positive, SOFA-1 only, SOFA-2 only, and concordant negative groups

Adult patients (age ≥ 18 years) with suspected infection were eligible for inclusion. The cohort selection process and group definitions are illustrated in Fig. 1.

### SOFA score calculation and group definition

For each patient, SOFA-1 and SOFA-2 scores were operationalized in accordance with their respective consensus guidelines (summarized in eTables 1 and 2) and calculated at an hourly resolution from ICU admission onward. Missing physiological variables were imputed using the last observation carried forward approach; if no prior value was available, the corresponding SOFA subscores were assumed to be 0, in accordance with the SOFA criteria. Detailed descriptions of the SOFA-2 updates are provided in the eMethods.

#### Sepsis detection

According to the Sepsis-3 criteria [3, 4], sepsis was defined as suspected infection accompanied by an acute increase in the total SOFA score of ≥ 2 points within a window spanning from 48 h before to 24 h after the onset of suspected infection. The change in score was calculated as the current score minus the cumulative minimum score observed up to that time point within the suspected infection window. For patients without known pre-existing organ dysfunction, baseline SOFA was assumed to be 0.

#### Group definition

Based on sepsis detection results derived from the two SOFA scoring criteria, patients were stratified into four mutually exclusive groups: (1) Concordant Positive (detected by both SOFA-1 and SOFA-2); (2) SOFA-2 Only (detected by SOFA-2 only); (3) SOFA-1 Only (detected by SOFA-1 only); and (4) Concordant Negative (detected by neither score).

### Outcomes

The primary outcome was ICU mortality. Secondary outcomes included hospital mortality and 28-day survival. In addition, we evaluated diagnostic concordance and the timing of sepsis detection (the time at which the ∆SOFA ≥ 2 criterion was first met within the infection window).

Patients in the Concordant Positive group were classified into three subgroups based on the relative timing of sepsis detection by the two scoring criteria: “SOFA-2 Earlier”, “Simultaneous”, or “SOFA-1 Earlier”. Given that the SOFA score was calculated at hourly resolution, simultaneous was defined as detection within the same hour by both scoring systems.

### Statistical analysis and predictive modeling

Continuous variables were summarized as medians with interquartile ranges (IQRs) or means with standard deviations (SDs), and compared using the Kruskal-Wallis test, as the Shapiro-Wilk test indicated non-normal distributions. Categorical variables were reported as frequencies and compared using the chi-square test. Overall estimates were calculated by pooling data across databases. Survival outcomes were visualized using Kaplan–Meier curves and compared via the log-rank test. Multivariable Cox proportional hazards models were fitted to estimate the hazard ratio (HR) for mortality. Models within each database were adjusted for age and sex, whereas the pooled model was additionally adjusted for database to account for heterogeneity across databases. A 2-sided *P* value *< .*05 was considered statistically significant. All statistical analyses were performed using Python (v3.12) with the packages SciPy (v1.14), lifelines (v0.29), and scikit-learn (v1.5).

#### **Organ-specific contribution analysis**

To identify which organ systems drove the diagnostic discordance, we performed an organ-level contribution analysis. For each patient in the SOFA-2 Only group, we examined why SOFA-2 detected sepsis while SOFA-1 did not. At the time of SOFA-2 diagnosis, we calculated the difference in acute dysfunction scores for each organ system between the two criteria: ∆diff_organ_ = ∆SOFA-2_organ_ − ∆SOFA-1_organ_. An organ was considered a contributor to the detection discordance if Δdiff_organ_ > 0, indicating that SOFA-2 scored that organ’s acute dysfunction higher than SOFA-1. For example, for renal dysfunction, we calculated Δdiff_renal_ as ΔSOFA-2_renal_ − ΔSOFA-1_renal_ at the time of SOFA-2 diagnosis; if Δdiff_renal_ > 0, renal dysfunction was considered to have contributed to the discordance in sepsis detection. We then calculated the proportion of patients in whom each organ system contributed to the detection difference. For the SOFA-1 Only group, the same approach was applied in the reverse direction at the time of SOFA-1 diagnosis.

#### Predictive modeling

To further compare the prognostic value of SOFA-1 and SOFA-2 in sepsis patients, we evaluated the predictive performance of machine learning models in forecasting ICU mortality among sepsis cohorts defined by the respective criteria [28–31]. We included all patients meeting sepsis criteria by either scoring system, with the selected time windows and SOFA trajectories described in detail in the eMethods. Model development followed a development–external validation framework. The training cohort comprised all sepsis-positive patients from MIMIC-IV and AmsterdamUMCdb (combined *n* = 29,241). Within this cohort, five machine learning models (Logistic Regression, Random Forest, Gradient Boosting, XGBoost, and LightGBM) were trained and internally validated using 5-fold stratified cross-validation. The best-performing model was selected based on the highest mean AUROC in cross-validation. External validation was performed on the independent multicenter eICU-CRD cohort (*n* = 12,209), which was held out entirely and was not used during model development or selection. The complete modeling pipeline is illustrated in eFigure 1. Performance was assessed using the Area Under the Receiver Operating Characteristic Curve (AUROC), and pairwise AUROC comparisons between SOFA-1 and SOFA-2 models were performed using the DeLong test [32]. Feature importance was analyzed using SHAP (SHapley Additive exPlanations) values [33]. Model calibration was evaluated using calibration curves and Brier scores.

## Results

### Diagnostic concordance between SOFA-1 and SOFA-2

**Fig. 2:**
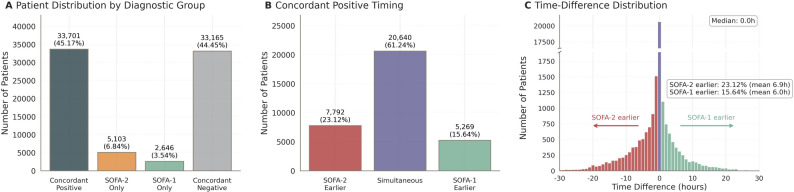
Diagnostic concordance and timing differences between SOFA-2 and SOFA-1 across three databases. (**A**) Distribution of diagnostic concordance groups based on SOFA-1 and SOFA-2 sepsis detection. (**B**) Timing-based subgroups within Concordant Positive patients (SOFA-1 earlier, simultaneous, or SOFA-2 earlier; simultaneity defined as detection within the same hour). (**C**) Distribution of diagnostic time differences among concordant positive patients, calculated as the SOFA-2 detection time minus the SOFA-1 detection time

The study cohort comprised 74,615 adult patients with suspected infection (MIMIC-IV: 48,460; eICU-CRD: 19,384; AmsterdamUMCdb: 6,771). Diagnostic overlap between SOFA-1 and SOFA-2 is illustrated in Fig. 2A. The majority (89.62%) of patients were classified consistently by both scores (Concordant Positive: 45.17%; Concordant Negative: 44.45%). SOFA-2 detected 5,103 additional patients (6.84%, the “SOFA-2 Only” group) who were missed by SOFA-1. Conversely, SOFA-1 detected 2,646 additional patients (3.54%, the “SOFA-1 Only” group) missed by SOFA-2. Despite differences in region and case composition, this distributional pattern was consistent across all three databases (eFig. 2A A, and 4 A).

Baseline characteristics were summarized over the 24 h following the diagnosis of sepsis with either SOFA-1 or SOFA-2. Compared with SOFA-2 Only and SOFA-1 Only groups, the Concordant Positive group had the highest lactate levels (2.32 vs. 1.80 vs. 1.82 mmol/L, *P* = 7.76 × 10^–41^), mechanical ventilation rates (55.4% vs. 38.6% vs. 32.4%, *P* = 4.20 × 10^–103^), vasopressor use (27.0% vs. 14.8% vs. 9.8%, *P* = 1.69 × 10^–74^), and ICU length of stay (6.59 vs. 5.74 vs. 5.54 days, *P* = 2.95 × 10^–17^) in MIMIC-IV, as detailed in eTable 3. Similar patterns were observed in eICU-CRD and AmsterdamUMCdb, as detailed in eTables 4 and 5.

### Comparison of diagnostic timing in concordant cases

Because SOFA-1 and SOFA-2 showed high diagnostic concordance, we next examined whether they differed in the timing of sepsis diagnosis. Among the 33,701 concordant positive patients, we analyzed the temporal alignment of sepsis diagnosis between the two scoring systems (Fig. 2B and C). Overall, SOFA-2 achieved earlier diagnosis in 23.12% of cases, whereas SOFA-1 preceded SOFA-2 in 15.64% of cases; the remaining 61.24% were diagnosed simultaneously. Database-specific analyses showed that SOFA-2 identified sepsis earlier than SOFA-1 more frequently in MIMIC-IV and eICU-CRD (MIMIC-IV: 25.67% vs. 14.94%; eICU-CRD: 20.68% vs. 16.98%), whereas the two scoring systems showed comparable early detection frequencies in AmsterdamUMCdb (15.79% vs. 16.24%). To compare the extent of earlier detection, we calculated the mean lead time and found that it was similar between the two scores (eFigs. 2 C, 3 C, and 4 C).

### Organ-specific contributors to diagnostic discordance

**Fig. 3:**
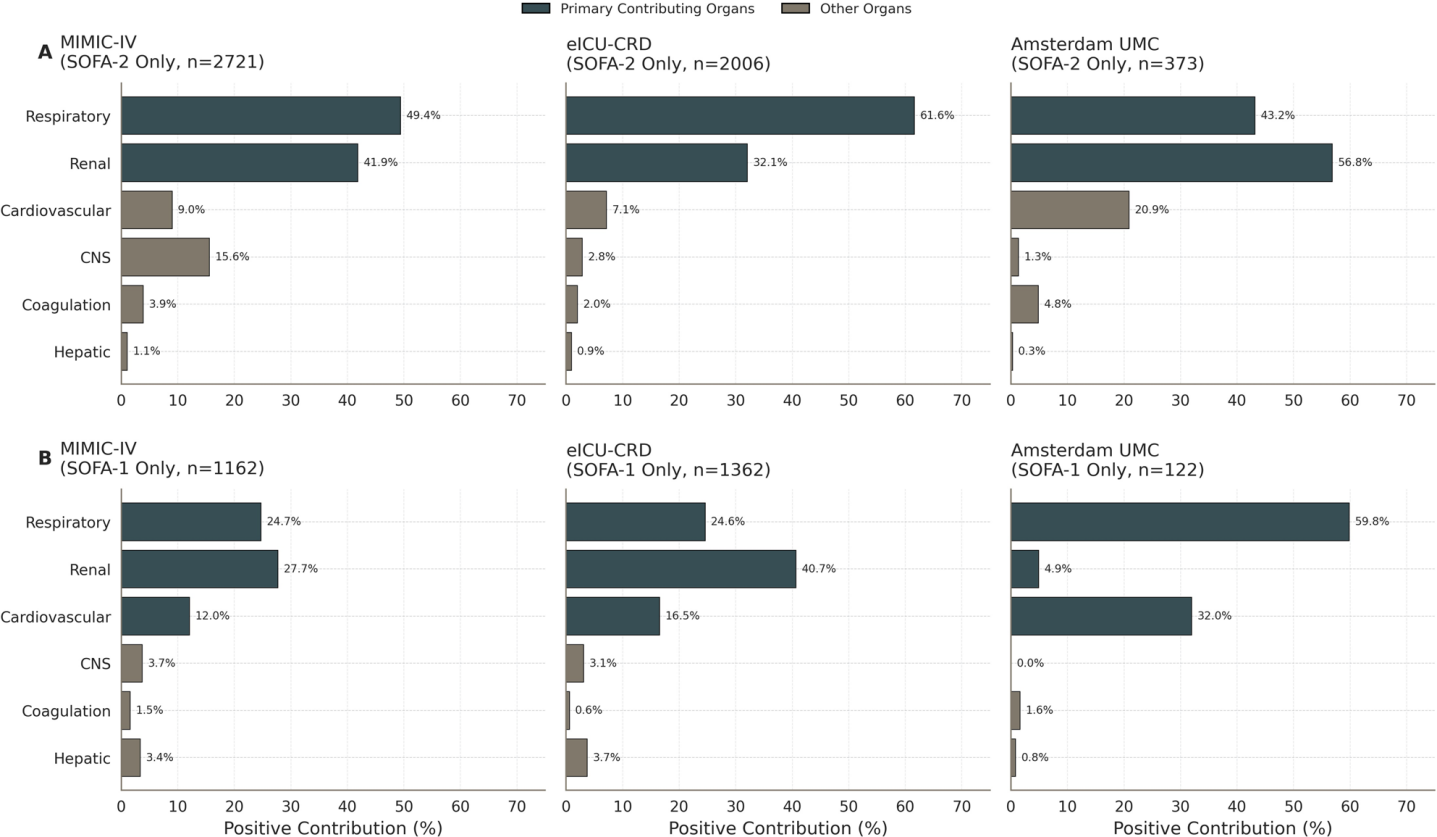
Organ system contributors to discordant detection. (**A**) Bars show the proportion of contributors within the SOFA-2 Only group across three databases. (**B**) Bars show contributors within the SOFA-1 Only group. Percentages sum to > 100% because a single patient may have contributions from multiple organs

To explore the determinants of diagnostic discordance, we analyzed organ-level contributions to diagnostic divergence (Fig. 3). In the SOFA-2 Only group, respiratory and renal systems were the predominant contributors (Fig. 3A), with respiratory dysfunction accounting for 43.2%–61.6% and renal dysfunction for 32.1%–56.8% of diagnostic differences across databases. For the respiratory system, SOFA-2 introduced SpO_2_/FiO_2_ ratios and high-flow nasal cannula (HFNC) and NIV support: patients meeting SpO_2_/FiO_2_ substitution criteria were predominantly observed in US databases (MIMIC-IV: 51.5%; eICU-CRD: 55.8%; eTable 7), with HFNC/NIV utilization rates of 49.4%–87.1%. For the renal system, SOFA-2 adopts weight-standardized urine output thresholds and shorter assessment windows, improving sensitivity to detect oliguria, particularly in patients with higher body weight.

In the SOFA-1 Only group, the respiratory, renal, and cardiovascular systems mainly contributed to the diagnostic divergence (Fig. 3B). For the respiratory system, advanced support utilization (44.2%–69.7%) was lower than in the SOFA-2 Only group (58.4%–87.7%), reflecting a scoring difference: SOFA-1 allows higher respiratory scores to be assigned based solely on PaO_2_/FiO_2_ impairment, whereas SOFA-2 requires the simultaneous use of advanced respiratory support to assign scores ≥ 3 (eTable 8). For the renal system, 7.6%–24.1% of patients met SOFA-1’s absolute oliguria threshold, whereas only 3.8%–10.0% met SOFA-2’s weight-standardized threshold (eTable 8). This difference reflects the change from absolute to weight-standardized urine output thresholds between SOFA-1 and SOFA-2. For the cardiovascular system, vasopressor usage ranged from 21.9% to 58.2%; among patients receiving norepinephrine, 54%–74% received doses ≤ 0.1 µg/kg/min, scoring 3 points under SOFA-1 but only 2 points under SOFA-2 (eTable 8).

### Comparative survival analysis of diagnostic groups

**Fig. 4:**
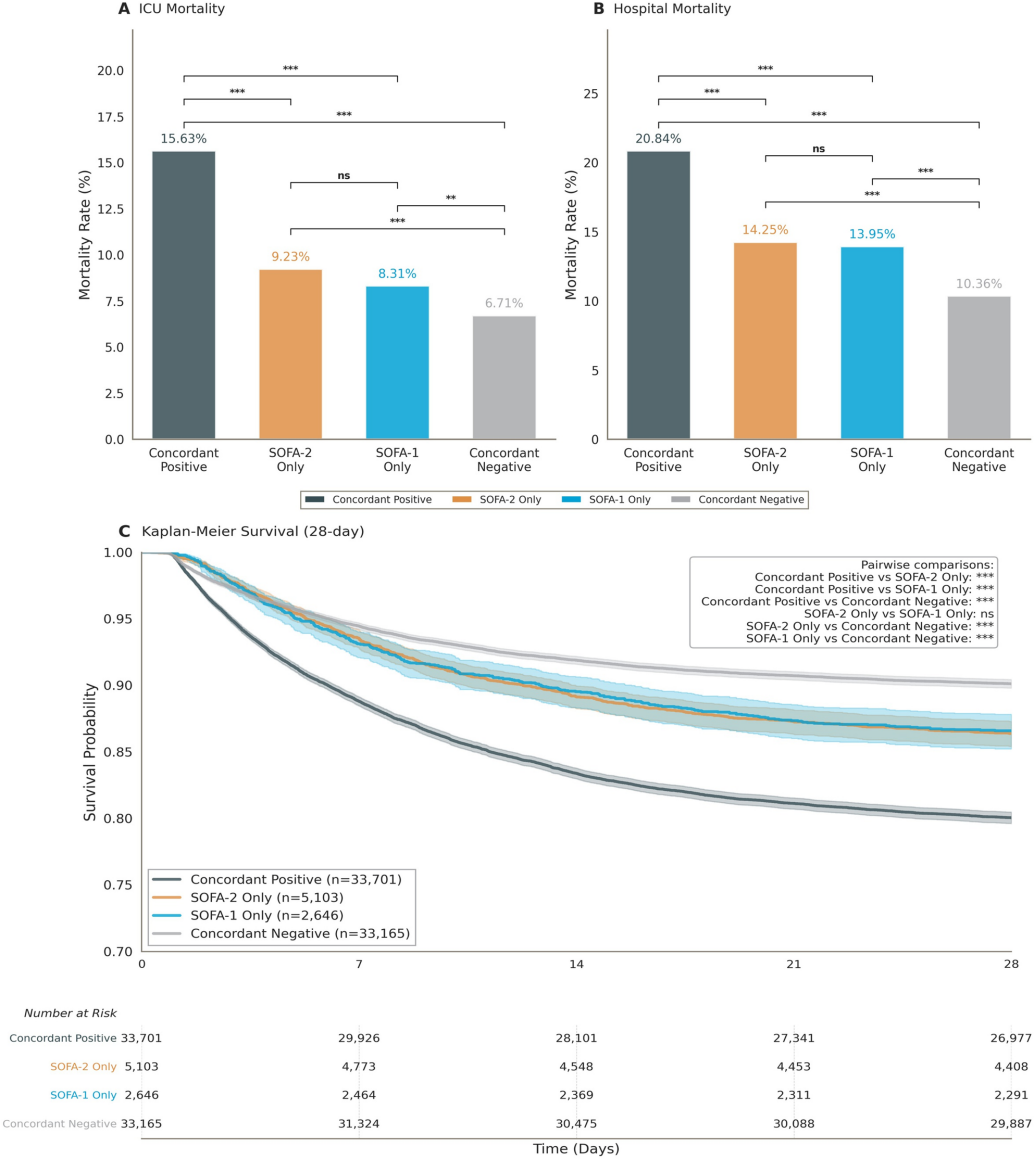
Clinical outcomes by diagnostic concordance group. (**A**) ICU mortality across four groups. (**B**) Hospital mortality in the pooled MIMIC-IV and eICU-CRD cohort, where hospital mortality was available. (**C**) Kaplan–Meier survival curves showing 28-day survival by group. ns indicates no significant difference; *** indicates *P* < .001; ** indicates *P* < .01; * indicates *P* < .05

To relate these discordant groups to patient outcomes, we examined ICU mortality as the primary outcome, along with hospital mortality and 28-day survival. In the pooled cohort across the three databases, the Concordant Positive group exhibited the highest ICU mortality (15.63%), followed by the SOFA-2 Only (9.23%) and SOFA-1 Only (8.31%), with the Concordant Negative group showing the lowest rate (6.71%). Pairwise comparisons showed that both discordant groups had higher ICU mortality than the Concordant Negative group (*P* = 2.87 × 10^–15^ and *P* = .002, respectively; Fig. 4A). In the pooled analysis of MIMIC-IV and eICU-CRD, where hospital mortality was available, the same pattern was observed, with rates of 20.84%, 14.25%, 13.95%, and 10.36% in the Concordant Positive, SOFA-2 Only, SOFA-1 Only, and Concordant Negative groups, respectively. Hospital mortality was similar between the SOFA-2 Only and SOFA-1 Only groups (*P* = .470; Fig. 4B), but both higher than the Concordant Negative group (both *P* < .001).

When stratified by database, both discordant groups in MIMIC-IV and eICU-CRD also showed higher ICU and hospital mortality than the Concordant Negative group (all *P* < .001). In AmsterdamUMCdb, where hospital mortality was unavailable, ICU mortality did not differ significantly between either discordant group and the Concordant Negative group (*P* = .230 for SOFA-2 Only and *P* = .374 for SOFA-1 Only). Notably, the AmsterdamUMCdb cohort exhibited a significantly longer ICU length of stay than the other two cohorts (eTables 3–5). Consequently, by focusing on short-term survival within the first 7 days post-diagnosis, a window that more closely reflects prognostic influence of diagnostic criteria, we observed concordant mortality patterns with the pooled cohort analysis, particularly for the SOFA-2 Only group (10.5% vs. 6.8%; *P* = .018; eFig. 7).

In the pooled 28-day Kaplan–Meier analysis across the three databases (Fig. 4C), the discordant groups showed intermediate survival between the Concordant Positive and Concordant Negative groups. No significant difference in 28-day survival was observed between the SOFA-2 Only and SOFA-1 Only groups. Database-specific survival analyses are shown in eFigs. 5, 6 and 7.

Using the SOFA-2 Only group as the reference, database-specific multivariable Cox models showed that Concordant Positive patients had significantly higher mortality risk in all three databases, whereas the SOFA-1 Only group showed no statistically significant difference in mortality risk compared with the SOFA-2 Only group in any database (eFigs. 8–10). A separate pooled Cox model adjusting for age, sex, and database showed the same pattern (eTable 9).

### Prognostic performance between SOFA-1 and SOFA-2

**Fig. 5:**
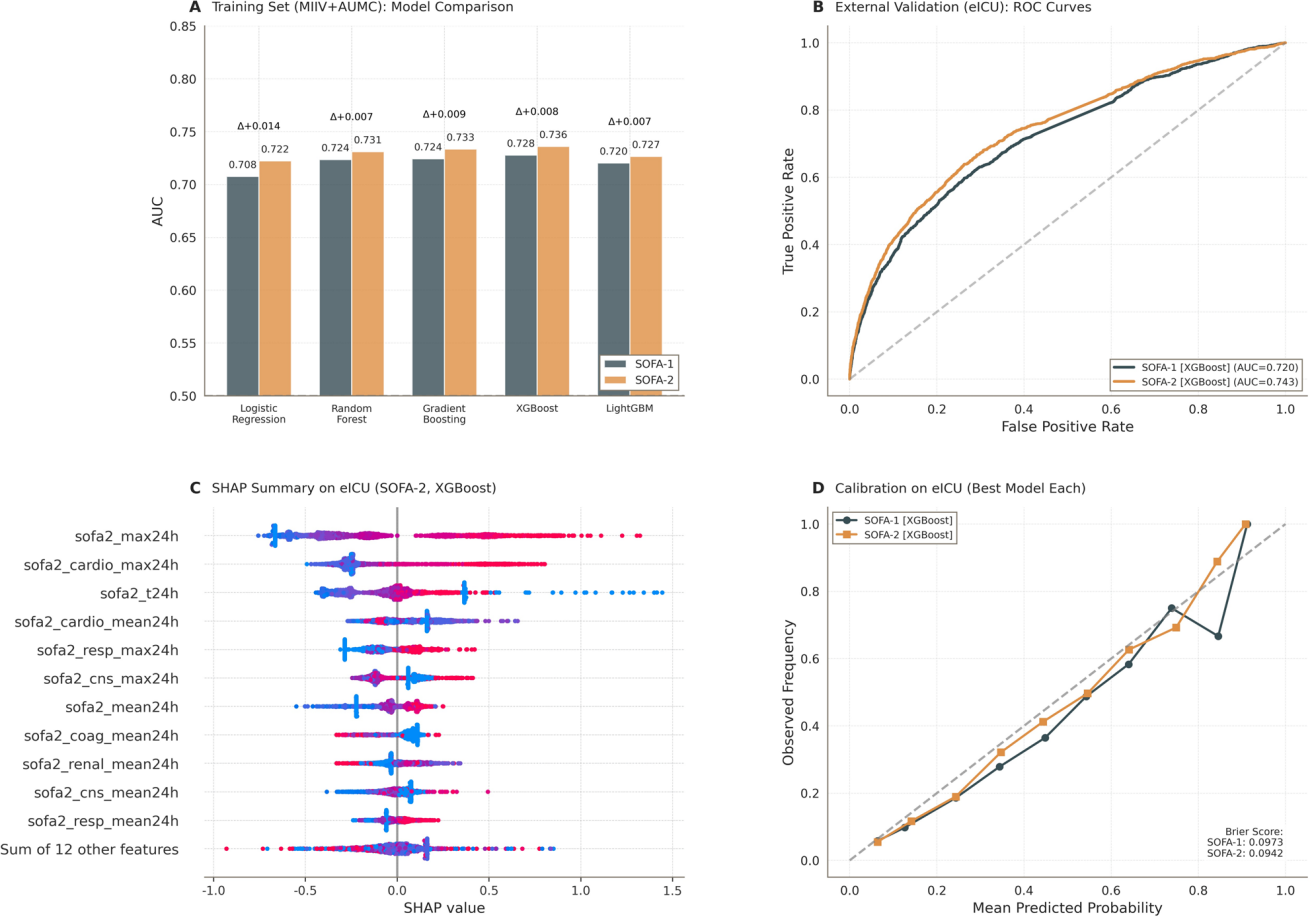
Cross-database performance of SOFA-based machine learning models for ICU mortality prediction. (**A**) Comparison of AUROC values across five machine learning models using SOFA-1 and SOFA-2 scores. (**B**) ROC curves comparing SOFA-1 and SOFA-2 using the best-performing model. (**C**) SHAP value summary plot for the best-performing SOFA-2 model in external validation. (**D**) Calibration curves comparing SOFA-1 and SOFA-2 using the best-performing model in the external validation cohort. Abbreviations used in panel labels: MIIV, MIMIC-IV; AUMC, AmsterdamUMCdb; eICU, eICU-CRD

The SOFA score serves as a continuous measure of disease severity and has value for prognostic assessment. To evaluate whether the updated criteria enhance this predictive capability in sepsis patients, we conducted a comparative evaluation of five machine learning models based on dynamic SOFA-1 and SOFA-2 scores for ICU mortality prediction, with dynamic score trajectories illustrated in eFigs. 12, 13 and 14. In internal cross-validation, XGBoost yielded the best performance for both scoring systems, with an AUROC of 0.736 (95% CI 0.727–0.743) for SOFA-2 and 0.728 (95% CI 0.717–0.734) for SOFA-1 (DeLong *P* = .386; Fig. 5A). In the external validation cohort, SOFA-2 demonstrated a numerically higher AUROC (0.743, 95% CI 0.729–0.757) than SOFA-1 (0.720, 95% CI 0.710–0.738), although this difference was not statistically significant (DeLong *P* = .375; Fig. 5B). The predictive performance of each dataset is presented in eFigs. 15, 16 and 17.

Feature importance analysis using SHAP revealed that cardiovascular and respiratory SOFA subscores contributed strongly to mortality prediction (Fig. 5C, eFigure 18). Calibration analysis demonstrated that the SOFA-2-based model not only provided reliable risk estimates across the entire probability range but also achieved better calibration than the SOFA-1-based model, with a lower Brier score (0.0942 vs. 0.0973; Fig. 5D), indicating improved alignment between predicted and observed risk.

## Discussion

In this multicenter study, SOFA-1 and SOFA-2 demonstrated high concordance in sepsis detection, yet each identified clinically meaningful discordant subgroups whose mortality was significantly higher than that of patients detected by neither score. The observed differences in sepsis detection between SOFA-1 and SOFA-2 were primarily attributable to updates in the respiratory and renal components. In particular, SOFA-2 incorporated SpO_2_/FiO_2_-based respiratory assessment as a surrogate for PaO_2_/FiO_2_, expanded the definition of advanced respiratory support to include HFNC and NIV, and revised criteria for mild respiratory dysfunction. In parallel, the renal component adopted weight-standardized urine output thresholds consistent with KDIGO criteria and assigned maximum scores for patients receiving renal replacement therapy [10, 12, 13, 34, 35]. Overall, these changes allowed SOFA-2 to identify additional patients missed by SOFA-1, and were associated with a higher proportion of earlier diagnosis in the pooled analysis and broadly comparable prognostic performance.

However, SOFA-1 also identified a clinically meaningful subgroup of patients with substantial mortality. This discordance may be partly due to differences in the updated renal and respiratory criteria. For the renal system, SOFA-2 assigns the maximum score to patients receiving RRT, a treatment decision that may vary across centers [36]. In addition, weight-standardized urine output thresholds may contribute to differences in renal classification [34]. For the respiratory system, the revised criteria for lower SOFA scores may reduce sensitivity for early respiratory failure, which has been associated with substantial mortality when under-recognized [36, 37]. Taken together, although SOFA-2 identified additional patients and showed favorable patterns in pooled diagnostic timing analyses, it did not fully overlap with the diagnostic coverage of SOFA-1. Accordingly, immediate and complete replacement of SOFA-1 may be premature, and a transitional strategy in which both scoring systems are calculated and reported may be more prudent.

Given that microbiology data in eICU-CRD were available from only 22 of 208 hospitals (10.6%) with electronic microbiology reporting systems, applying the standard culture-plus-antibiotics definition to the entire cohort was not feasible. Restricting the analysis to this small subset of hospitals would substantially compromise both statistical power and the multicenter representativeness. Therefore, consistent with prior multi-database studies [38–41], we adopted a pragmatic approach for eICU-CRD by identifying suspected infection using infection-related ICD codes together with antibiotic administration, whereas the standard culture-plus-antibiotics definition was used in MIMIC-IV and AmsterdamUMCdb. We then evaluated the validity of this methodological adaptation and its impact on sepsis diagnosis in MIMIC-IV database, which has both ICD and microbiology data. Our analysis demonstrated high concordance, with 87.4% of ICD-defined infections and 88.2% of ICD-defined sepsis cases also meeting the standard culture-plus-antibiotics definition (eFigure 19). This substantial overlap supports the ICD-based approach as a reasonable surrogate when microbiology records are unavailable. To ensure the robustness of our findings, we conducted a sensitivity analysis comparing our primary mixed-definition approach with the culture-based approach across the databases. The two definitions showed similar concordance patterns, diagnostic timing and mortality outcomes (eFigure 20), indicating that the divergence in infection definitions did not materially affect the main results.

Early sepsis recognition is clinically important, as prior studies have shown that each hour of delay in treatment is associated with a 4% to 9% relative increase in mortality risk [42, 43]. Among concordant patients, SOFA-2 achieved earlier identification in 23.12% of cases versus 15.64% for SOFA-1; however, it remains unproven whether this timing advantage translates into meaningful clinical benefit. Prospective studies are needed to evaluate its impact on patient outcomes.

Notably, the AmsterdamUMCdb cohort exhibited several divergent trends compared with the other two cohorts. Regarding diagnostic timing and prognostic model performance (AUROC), both MIMIC-IV and eICU-CRD demonstrated consistent trends toward earlier diagnosis and higher AUROC under SOFA-2. However, the lack of statistical significance in these findings implies that the divergent pattern in AmsterdamUMCdb does not constitute a fundamental contradiction of our primary findings. The most prominent divergence was observed in the survival analysis across the four diagnostic groups for ICU and 28-day mortality (eFigure 7a, c). However, when the analysis was confined to the first 7 days after diagnosis, mortality trajectories were consistent across all three databases (eFigure 7b, d). This temporal distinction is clinically meaningful, as short-term mortality is more likely to reflect the acute consequences of sepsis diagnostic criteria and early management. The longer-term discrepancies observed in AmsterdamUMCdb are likely caused by competing risks rather than the initial septic event, particularly given this population’s high prevalence of severe cardiovascular and respiratory comorbidities and a substantially greater proportion of post-surgical admissions [26]. Over longer follow-up periods, such as 28 days or the entire ICU stay, outcomes in this cohort may be more likely influenced by competing mortality risks. These risk factors may gradually attenuate or obscure the prognostic differences associated with the initial sepsis definitions. Additionally, the sample size of the discordant subgroups in AmsterdamUMCdb is nearly tenfold smaller compared to the MIMIC-IV and eICU-CRD cohorts. Such a disproportionately smaller sample size inherently diminishes the statistical power required to capture the significant prognostic divergences seen in the primary analysis.

### Limitations

This study has several limitations. First, although standardized data cleaning was applied, some records in this retrospective analysis may still be incomplete or erroneous, potentially affecting SOFA score calculation and sepsis detection. The completeness of SOFA-related variables varied across databases (eTable 10), with several variables required for SOFA-2 calculation being less complete in eICU-CRD than in MIMIC-IV and AmsterdamUMCdb. This reduced completeness may limit the ability of SOFA-2 to accurately capture organ dysfunction in eICU-CRD, thereby contributing to the lower concordance between SOFA-1 and SOFA-2 (eTable 6). Additionally, we assumed a baseline SOFA score of 0 for patients without documented pre-existing organ dysfunction, which is standard in retrospective studies but may overestimate the ΔSOFA in patients with chronic impairment [44]. Finally, suspected infection could not be defined identically across the three databases because microbiology data were incompletely captured in eICU-CRD. In this database, suspected infection was therefore identified using infection-related ICD codes together with antibiotic administration rather than the standard culture-plus-antibiotics definition. This approach may have introduced selection bias or misclassification by including some patients with less definite or milder infection, while excluding others who might have been identified under the culture-plus-antibiotics definition but were not appropriately coded. Prospective multicenter studies across diverse healthcare settings are needed to further validate and refine the clinical application of SOFA-2 [45].

## Conclusions

SOFA-1 and SOFA-2 showed high concordance in sepsis detection, yet each identified distinct patient subgroups whose mortality was substantially higher than that of patients detected by neither score. The diagnostic differences are primarily attributable to the updates in respiratory and renal scoring criteria. SOFA-2 achieved earlier diagnosis more frequently, yet prognostic models demonstrated comparable performance between the two scores. Therefore, a transitional strategy utilizing both SOFA-1 and SOFA-2 is advised until updated expert-validated sepsis criteria are established.

## Supplementary Information

Below is the link to the electronic supplementary material.

Supplementary Material 1.

## Data Availability

The data used in this study are publicly available from MIMIC-IV (https://physionet.org/content/mimiciv/), eICU-CRD (https://physionet.org/content/eicu-crd/), and AmsterdamUMCdb (https://amsterdammedicaldatascience.nl/) upon completion of required data use agreements and credentialing processes. The analysis code will be made available upon reasonable request to the corresponding author.

## Abbreviations

ARDS: Acute respiratory distress syndrome
AUROC: Area under the receiver operating characteristic curve
eICU-CRD: eICU Collaborative Research Database
FiO_2_: Fraction of inspired oxygen
HFNC: High-flow nasal cannula
HR: Hazard ratio
ICD: International Classification of Diseases
ICU: Intensive care unit
KDIGO: Kidney Disease: Improving Global Outcomes
LightGBM: Light Gradient Boosting Machine
MIMIC-IV: Medical Information Mart for Intensive Care IV
NIV: Non-invasive ventilation
PaO_2_/FiO_2_: Ratio of arterial oxygen partial pressure to fraction of inspired oxygen
RRT: Renal replacement therapy
Sepsis-3: Third International Consensus Definitions for Sepsis and Septic Shock
SHAP: SHapley Additive exPlanations
SOFA: Sequential Organ Failure Assessment
SpO_2_/FiO_2_: Ratio of peripheral oxygen saturation to fraction of inspired oxygen
STROBE: Strengthening the Reporting of Observational Studies in Epidemiology
XGBoost: Extreme Gradient Boosting
