# Supplementary material for: Impact of the updated SOFA-2 score on sepsis diagnosis and prognosis: a retrospective multicenter cohort study
Source: Crit Care. 2026 May 6;30:338. doi: 10.1186/s13054-026-06070-1 (PMC13312726; doi:10.1186/s13054-026-06070-1)
Supplement: Supplementary file 1 — Supplementary Material 1. [file 13054_2026_6070_MOESM1_ESM.docx]

Supplemental Online Content

**Impact of the Updated SOFA-2 Score on Sepsis Diagnosis and Prognosis: A Retrospective Multicenter Cohort Study**

Haibo Zhu; Peirong Li; Bing Wang; Hao Fu; Yehan Guo; Ziyi Han; Shuojing Huang; Yujie Xie;

Jun He; Shixiang Zheng; Xiaopei Shen

# Contents

[Contents 1](#_Tocz8axl2)

[eMethods 1](#_Tocf30wz2)

[eTable 1. SOFA-1 Scoring Criteria 4](#_Toczvvhzq)

[eTable 2. SOFA-2 Scoring Criteria 5](#_Tocyddqfw)

[eTable 3. Detailed Baseline Characteristics by Diagnostic Group (MIMIC-IV) 7](#_Tocxpv9ju)

[eTable 4. Detailed Baseline Characteristics by Diagnostic Group (eICU-CRD) 9](#_Toc361ka2)

[eTable 5. Detailed Baseline Characteristics by Diagnostic Group (AmsterdamUMCdb) 11](#_Tochyd43o)

[eTable 6. Clinical Severity and Organ Support Utilization Among Sepsis-Positive Patients 13](#_Toctgth5b)

[eTable 7. SOFA-2 Only Group: Clinical Characteristics at Sepsis Diagnosis 14](#_Tocu85jwp)

[eTable 8. SOFA-1 Only Group: Clinical Characteristics at Sepsis Diagnosis 16](#_Tocalxp8x)

[eTable 9. Pooled Cox Model for 28-Day Mortality Adjusted for Age, Sex, and Database 18](#_Tocgb26xa)

[eTable 10. Proportion of Missing Data for SOFA Component Variables 19](#_Toc0y3xfn)

[eFigure 1. Predictive Modeling Development and Validation Framework 20](#_Toc1cjsoo)

[eFigure 2. Diagnostic Concordance and Timeliness Between SOFA-2 and SOFA-1 (MIMIC-IV) 21](#_Toc7wec2t)

[eFigure 3. Diagnostic Concordance and Timeliness Between SOFA-2 and SOFA-1 (eICU-CRD) 22](#_Toczqsy4c)

[eFigure 4. Diagnostic Concordance and Timeliness Between SOFA-2 and SOFA-1 (AmsterdamUMCdb) 23](#_Tocwtncmm)

[eFigure 5. Clinical Outcomes by Diagnostic Concordance Group (MIMIC-IV) 24](#_Toc30wkyz)

[eFigure 6. Clinical Outcomes by Diagnostic Concordance Group (eICU-CRD) 25](#_Tocob33mz)

[eFigure 7. Clinical Outcomes by Diagnostic Concordance Group (AmsterdamUMCdb) 26](#_Tocz6y3wq)

[eFigure 8. Cox Regression Forest Plot (MIMIC-IV) 27](#_Toc7o4rm3)

[eFigure 9. Cox Regression Forest Plot (eICU-CRD) 28](#_Toc2gjhho)

[eFigure 10. Cox Regression Forest Plot (AmsterdamUMCdb) 29](#_Tocs43q9e)

[eFigure 11. Dumbbell Plot of SOFA-1 vs SOFA-2 Organ Score Differences 30](#_Tocgv1nvu)

[eFigure 12. Dynamic SOFA Trajectories (MIMIC-IV) 31](#_Tocl053oy)

[eFigure 13. Dynamic SOFA Trajectories (eICU-CRD) 32](#_Tocscq25l)

[eFigure 14. Dynamic SOFA Trajectories (AmsterdamUMCdb) 33](#_Tocdz1w6t)

[eFigure 15. Machine Learning Model Comparison (MIMIC-IV) 34](#_Toca20kau)

[eFigure 16. Machine Learning Model Comparison (eICU-CRD) 35](#_Tocj3z1pq)

[eFigure 17. Machine Learning Model Comparison (AmsterdamUMCdb) 36](#_Toc4vn1kn)

[eFigure 18. SHAP Summary Plot for SOFA-1 Features 36](#_Tocojx5n6)

[eFigure 19. Overlap Between Infection and Sepsis Definitions in MIMIC-IV 38](#_Tocebk433)

[eFigure 20. Sensitivity Analysis Comparing Mixed-Definition and Culture-Based Suspected Infection Definitions 39](#_Tock93hwu)

[References 40](#_Toc0nwcrg)

*This supplemental material has been provided by the authors to give readers additional information about their work.*

eMethods

**1. Database-Specific Preprocessing**

Given substantial differences in data architecture, variable organization, and recording granularity across the three databases, database-specific preprocessing was conducted prior to score derivation. Although all datasets contained the variables required to compute both SOFA-1 and SOFA-2 components, heterogeneity in data structure and completeness necessitated tailored harmonization before applying a unified scoring pipeline.

For each database, raw data were first mapped into a common analytical structure by harmonizing source tables and variable definitions, standardizing measurement units, and aligning all time-stamped observations to a unified hourly time grid. Implausible values were excluded using predefined physiologic range checks. When multiple measurements were recorded within the same hour, values were aggregated according to prespecified rules.

Urine output was normalized to the time windows required for renal SOFA assessment, and respiratory support status was derived from treatment and charting data using a consistent classification framework across databases. Following these preprocessing steps, SOFA-1 and SOFA-2 scores were calculated using an identical downstream pipeline in all cohorts.

**2. Handling of Missing Data**

Missing physiological variables were imputed using the last observation carried forward (LOCF) method, consistent with the ricu framework. When no prior value was available, the corresponding SOFA subscore was assumed to be 0, in accordance with both the original SOFA and SOFA-2 consensus recommendations. The proportion of missing values for each SOFA component at sepsis diagnosis in each database is reported in eTable 10.

**3. Observation Window Standardization**

To ensure comparability across databases with different data structures and recording characteristics, we restricted sepsis detection to the first 72 hours following ICU admission. This standardized observation window was necessary because the databases differ in their data recording scope: MIMIC-IV [1] includes comprehensive data from emergency department and hospital ward encounters prior to ICU admission through the hospital-wide electronic health record; eICU-CRD [2] captures clinical data starting around the time of ICU admission with variable pre-ICU laboratory data availability depending on hospital-specific data interfaces; and AmsterdamUMCdb [3] primarily records data from ICU admission with limited pre-ICU clinical measurements. By applying a consistent 72-hour post-admission window for sepsis detection, we ensured that comparisons between SOFA-1 [4] and SOFA-2 [5] were based on comparable observation periods across all three databases.

**4. Definition of Suspected Infection**

Suspected infection was identified by the temporal co-occurrence of clinical evidence of infection and administration of systemic antibiotics. In MIMIC-IV and AmsterdamUMCdb, clinical evidence of infection was defined as body fluid sampling for culture, with the infection window spanning antibiotics administered within 72 hours after culture or cultures obtained within 24 hours after antibiotics [6]. In eICU-CRD, due to limited microbiology data availability, clinical evidence of infection was instead identified using infection-related ICD diagnosis codes, consistent with prior surveillance methodology using administrative data [7–10]. In all three databases, the timing of suspected infection onset was anchored to the initiation of systemic antibiotics.

**5. SOFA Score Calculation**

The SOFA-1 score was computed according to the original 1996 criteria (eTable 1[).](file:///C:\Users\Dell\Downloads\cc7xle#_bookmark5) The SOFA-2 score was computed according to the updated 2025 [criteria,](file:///C:\Users\Dell\Downloads\8og1v1#_bookmark8) incorporating the following key modifications: (1) for the respiratory system, lowered PaO_2_/FiO_2_ thresholds, use of SpO_2_/FiO_2_ ratios when PaO_2_ was unavailable and SpO_2_ was <98%, and inclusion of advanced ventilatory support (including high-flow nasal cannula and noninvasive ventilation) in scoring thresholds; (2) for the cardiovascular system, summation of norepinephrine and epinephrine doses and incorporation of mechanical circulatory support; (3) for the renal system, use of weight-standardized urine output thresholds assessed over 6-, 12-, and 24-hour windows; and (4) for the neurological system, use of pre-sedation Glasgow Coma Scale scores and awarding 1 point for patients receiving delirium treatment; updated cutoffs were also applied for coagulation and liver components as specified in the consensus criteria (eTable 2).

**6. Calculation of ∆SOFA**

Consistent with the Sepsis-3 criteria, the ∆SOFA for each organ was calculated as the difference between the current subscore and the cumulative minimum subscore observed up to that time point, reflecting the acute increase from the patient’s individual baseline

**7. Sepsis-3 Event Labeling**

Sepsis-3 [6] events were detected from hourly SOFA trajectories and SI events using the procedure consistent with *ricu* [11]. The following algorithm was applied separately to the time series data computed from SOFA-1 and SOFA-2 scores:

- An SI time window was constructed as [SI*−*48h, SI+24h].
- Within the SI window, ∆SOFA was computed as current SOFA score minus the cumulative minimum SOFA score observed up to that time point within the suspected infection window.
- A Sepsis-3 event was detected at the earliest hour where ∆SOFA *≥* 2.
- Only the first Sepsis-3 event per stay was retained.

**8. Organ-Specific Contribution Analysis**

For each patient in the SOFA-2 Only group, we computed the organ-level delta difference as: ∆diff_organ_ = ∆SOFA-2_organ_ *−* ∆SOFA-1_organ_ at the time of SOFA-2 diagnosis. A positive delta difference indicates that the organ’s acute dysfunction was scored higher under SOFA-2 than under SOFA-1. We calculated the proportion of patients where each organ system contributed to the detection difference. For the SOFA-1 Only group, we applied the reverse calculation ∆diff_organ_ = ∆SOFA-1_organ_ *−* ∆SOFA-2_organ_ at the time of SOFA-1 diagnosis to identify organs driving SOFA-1–specific identification.

**9. Time windows and SOFA trajectories in Predictive Modeling**

A unified temporal reference point was established for each patient: for Concordant Positive patients, the earlier of the two diagnosis times was used; for discordant groups, the respective diagnosis time (SOFA-1 or SOFA-2) was used. Models were trained on the same patient population using identical temporal reference points. Dynamic SOFA scores were extracted from the first 24 hours following this unified diagnosis time. Thus, the only difference between the two models was the scoring system itself, enabling a direct and fair comparison of their prognostic performance. Only patients with at least 24 hours of post-diagnosis data were included in the analysis. Features were extracted at 6-hour intervals to characterize the dynamic trajectory of organ dysfunction over the first 24 hours.

eTable 1. SOFA-1 Scoring Criteria

| **Organ system** | **0** | **1** | **2** | **3** | **4** |
| --- | --- | --- | --- | --- | --- |
| Respiratory | PaO_2_/FiO_2_ > 400 mm Hg | PaO_2_/FiO_2_ ≤ 400 mm Hg | PaO_2_/FiO_2_ ≤ 300 mm Hg | PaO_2_/FiO_2_ ≤ 200 mm Hg with respiratory support | PaO_2_/FiO_2_ ≤ 100 mm Hg with respiratory support |
| Cardiovascular | MAP ≥ 70 mm Hg | MAP < 70 mm Hg | Dopamine ≤ 5 μg/kg/min or dobutamine (any dose) | Dopamine > 5 μg/kg/min or epinephrine ≤ 0.1 μg/kg/min or norepinephrine ≤ 0.1 μg/kg/min | Dopamine > 15 μg/kg/min or epinephrine > 0.1 μg/kg/min or norepinephrine > 0.1 μg/kg/min |
| Renal | Creatinine < 1.2 mg/dL | Creatinine 1.2–1.9 mg/dL | Creatinine 2.0–3.4 mg/dL | Creatinine 3.5–4.9 mg/dL or urine output < 500 mL/day^b^ | Creatinine > 5.0 mg/dL or urine output < 200 mL/day^a^ |
| CNS | GCS 15 | GCS 13–14 | GCS 10–12 | GCS 6–9 | GCS < 6 |
| Liver | Bilirubin < 1.2 mg/dL | Bilirubin 1.2–1.9 mg/dL | Bilirubin 2.0–5.9 mg/dL | Bilirubin 6.0–11.9 mg/dL | Bilirubin ≥ 12.0 mg/dL |
| Coagulation | Platelets ≥ 150×10³/μL | Platelets < 150×10³/μL | Platelets < 100×10³/μL | Platelets < 50×10³/μL | Platelets < 20×10³/μL |

Abbreviations: GCS, Glasgow Coma Scale; MAP, mean arterial pressure; PaO2/FiO2, ratio of partial pressure of oxygen to fraction of inspired oxygen.

SOFA-1 uses absolute urine output thresholds (mL/day), whereas SOFA-2 uses weight-standardized thresholds (mL/kg/h) assessed over 6-, 12-, and 24-hour windows.

eTable 2. SOFA-2 Scoring Criteria

| Organ system | Score 0 | Score 1 | Score 2 | Score 3 | Score 4 |
| --- | --- | --- | --- | --- | --- |
| Brain^a, b^ | GCS 15 (or thumbs-up, fist, or peace sign) | GCS 13–14 (or localizing to pain)^b^ or need for drugs to treat delirium^c^ | GCS 9–12 (or withdrawal to pain) | GCS 6–8 (or flexion to pain) | GCS 3–5 (or extension to pain, no response to pain, generalized myoclonus) |
| Respiratory^d^ | PaO_2_/FiO_2_ >300 mm Hg (>40 kPa) | PaO_2_/FiO_2_≤300 mm Hg (≤40 kPa) | PaO_2_/FiO_2_ ≤225 mm Hg (≤30 kPa) | PaO_2_/FiO_2_ ≤150 mm Hg (≤20 kPa) and advanced ventilatory support^e,f^ | PaO_2_/FiO_2_ ≤75 mm Hg (≤10 kPa) and advanced ventilatory support or ECMO^g^ |
| Cardiovascular^h,I,j,k^ | MAP ≥70 mm Hg, no vasopressor or inotrope | MAP <70 mm Hg, no vasopressor or inotrope | Low-dose vasopressor (norepinephrine + epinephrine ≤0.2 μg/kg/min) or any dose of other vasopressor or inotrope | Medium-dose vasopressor (norepinephrine + epinephrine >0.2 to ≤0.4 μg/kg/min) or low-dose vasopressor with any other vasopressor or inotrope | High-dose vasopressor (norepinephrine + epinephrine >0.4 μg/kg/min) or medium-dose vasopressor with any other vasopressor or inotrope or mechanical support^g,l^ |
| Liver | Total bilirubin ≤1.20 mg/dL | Total bilirubin ≤3.0 mg/dL | Total bilirubin ≤6.0 mg/dL | Total bilirubin ≤12.0 mg/dL | Total bilirubin >12 mg/dL |
| Kidney | Creatinine ≤1.20 mg/dL | Creatinine ≤2.0 mg/dL or urine output <0.5 mL/kg/h (6–12 h) | Creatinine ≤3.50 mg/dL or urine output <0.5 mL/kg/h (≥12 h) | Creatinine >3.50 mg/dL or urine output <0.3 mL/kg/h (≥24 h) or anuria (0 mL) for ≥12 h | Receiving or meeting criteria for RRT (includes chronic use)^m,n,o^ |
| Hemostasis | Platelets >150 ×10³/μL | Platelets ≤150 ×10³/μL | Platelets ≤100 ×10³/μL | Platelets ≤80 ×10³/μL | Platelets ≤50 ×10³/μL |

Abbreviations: ECMO, extracorporeal membrane oxygenation; GCS, Glasgow Coma Scale; MAP, mean arterial pressure; PaO_2_:FiO_2_, ratio of partial pressure of oxygen to fraction of inspired oxygen; RRT, renal replacement therapy; SOFA, Sequential Organ Failure Assessment.

The final score is obtained by summing the maximum points from each of the 6 organ systems individually within a 24-hour period, ranging from 0 to 24. For missing values at day 1, the general recommendation is to score these as 0 points. This may vary for specific purposes (eg, bedside use, research, etc). For sequential scoring, for missing data after day 1, it is to carry forward the last observation, the rationale being that nonmeasurement suggests stability.

a For sedated patients, use the last recorded GCS before sedation. If the previous GCS is unknown, score 0.

b When not possible to evaluate the 3 domains of GCS, use the best achieved score in the motor-scale domain.

c If receiving drug treatment for delirium, score 1 point even if GCS is 15. For relevant drugs, see the International Management of Pain, Agitation, and Delirium in Adult Patients in the ICU Guidelines.

d Use the arterial oxygen saturation (SpO_2_) to FiO_2_ ratio only when the PaO_2_:FiO_2_ ratio is unavailable and when the SpO_2_ is less than 98%. Cutoffs: 0 points, greater than 300 mm Hg; 1 point, 300 mm Hg or less; 2 points, 250 mm Hg or less; 3 points, 200 mm Hg or less with ventilatory support; 4 points, 120 mm Hg or less with ventilatory support or ECMO.

e Advanced ventilatory support is defined as receipt of high-flow nasal cannula, continuous positive airflow pressure, bilevel positive airway pressure, noninvasive ventilation, invasive mechanical ventilation, or long-term home ventilation. This is required to score 3 to 4 points, in addition to the PaO_2_:FiO_2_ or SpO_2_:FiO_2_ ratio being within the specified range. Changes in PaO_2_/FiO_2_ or SpO_2_/FiO_2_ within 1 hour (eg, after suctioning) should not be considered.

f Patients not receiving advanced respiratory support can score a maximum of 2 points unless ventilatory support is (1) not available or (2) precluded due to the ceiling of treatment; if so, severity is scored by the PaO_2_:FiO_2_ or SpO_2_:FiO_2_ ratio.

g If used for respiratory failure, ECMO (all forms) should be scored 4 in the respiratory component (regardless of PaO_2_:FiO_2_ ratio), but not in the cardiovascular component. If used for cardiovascular indications (all forms), it should be automatically scored in both the cardiovascular and the respiratory systems.

h Vasopressor medication is only scored if given by continuous intravenous infusion for at least 1 hour.

i Norepinephrine is usually dispensed as the salt (eg, hemitartrate or bitartrate). Dose should be expressed as the base. One mg of norepinephrinebase is equivalent to 2 mg of norepinephrine bitartrate monohydrate, 1.89 mg of the anhydrous bitartrate (also called hydrogen tartrate, acid tartrate, or tartrate), and 1.22 mg of the hydrochloride.

j If dopamine is used as a single vasopressor, scoring is based on the following cutoffs: 2 points (*≤*20 *µ*g/kg/min); 3 points (*>*20 to *≤*40 *µ*g/kg/min); 4 points (*>*40 *µ*g/kg/min). These cutoffs are based on norepinephrine equipotency studies.

k When vasoactive drugs are unavailable or precluded due to a ceiling of treatment, use the following MAP cutoffs for scoring: 0 point, 70 mm Hg or higher; 1 point, 60 to 69 mm Hg; 2 points, 50 to 59 mm Hg; 3 points, 40 to 49 mm Hg; 4 points, less than 40 mm Hg.

l Any type of mechanical cardiovascular support: eg, venoarterial ECMO, intra-aortic balloon pump, left ventricular assist device, microaxial flow pump.

m Excludes patients receiving RRT exclusively for nonrenal causes (eg, removal of toxic products, bacterial toxins, cytokines).

n For patients not receiving RRT (eg, ceiling of treatment, machine unavailability, or decision to delay commencement), score 4 points if they otherwise meet criteria for RRT, ie, creatinine level greater than 1.2 mg/dL (*>*110 *µ*mol/L) or oliguria (*<*0.3 mL/kg/h) for more than 6 hours plus at least 1 of either serum potassium of 6.0 mmol/L or greater or metabolic acidosis with pH of 7.20 or less and serum bicarbonate of 12 mmol/L or less.

o For patients receiving intermittent RRT, score 4 points on days not receiving RRT until RRT use is terminated.

eTable 3. Detailed Baseline Characteristics by Diagnostic Group (MIMIC-IV)

| **Characteristic** | **Category** | **Concordant Positive** | **SOFA-2 Only** | **SOFA-1 Only** | **P value** |
| --- | --- | --- | --- | --- | --- |
| No. of patients | Sample size | 20,635 | 2,724 | 1,162 |  |
| Age, y | Demographics | 63.65 ± 16.03 | 62.47 ± 16.67 | 62.90 ± 16.78 | .004 |
| Male sex | Demographics | 11,829 (57.3%) | 1,461 (53.6%) | 621 (53.4%) | <.001 |
| ICU length of stay, d (median) | Demographics | 4.00 (2.19–7.90) | 3.43 (2.01–6.54) | 3.46 (2.01–6.38) | <.001 |
| PaO_2_, mm Hg (mean) | SOFA-1 Respiratory | 111.66 ± 54.80 | 118.04 ± 58.25 | 95.44 ± 44.06 | <.001 |
| FiO_2_, % (mean) | SOFA-1 Respiratory | 53.02 ± 15.01 | 49.84 ± 16.07 | 50.00 ± 14.31 | <.001 |
| PaO_2_/FiO_2_ ratio (mean) | SOFA-1 Respiratory | 289.24 ± 157.44 | 330.01 ± 195.84 | 281.89 ± 169.65 | <.001 |
| Mechanical ventilation, any | SOFA-1 Respiratory | 11,442 (55.4%) | 1,052 (38.6%) | 376 (32.4%) | <.001 |
| SpO_2_, % (mean) | SOFA-2 Respiratory (updated) | 96.42 ± 2.10 | 96.13 ± 2.03 | 95.99 ± 2.02 | <.001 |
| SpO_2_/FiO_2_ ratio (mean) | SOFA-2 Respiratory (updated) | 198.79 ± 51.91 | 214.50 ± 55.73 | 210.05 ± 56.61 | <.001 |
| Advanced respiratory support, any | SOFA-2 Respiratory (updated) | 13,903 (67.4%) | 1,565 (57.5%) | 500 (43.0%) | <.001 |
| ECMO, any | SOFA-2 Respiratory (updated) | 78 (0.4%) | 8 (0.3%) | 1 (0.1%) | .23 |
| Platelets, ×10³/µL (mean) | SOFA-2 Coagulation | 193.17 ± 115.46 | 227.68 ± 123.74 | 227.69 ± 116.94 | <.001 |
| Total bilirubin, mg/dL (mean) | SOFA Liver | 2.66 ± 5.42 | 2.12 ± 5.10 | 2.22 ± 6.15 | <.001 |
| Mean arterial pressure, mm Hg (mean) | SOFA Cardiovascular (baseline) | 75.81 ± 9.81 | 77.86 ± 10.80 | 78.38 ± 11.41 | <.001 |
| Systolic blood pressure, mm Hg (mean) | SOFA Cardiovascular (baseline) | 114.27 ± 15.19 | 118.20 ± 16.12 | 116.75 ± 16.31 | <.001 |
| Diastolic blood pressure, mm Hg (mean) | SOFA Cardiovascular (baseline) | 60.80 ± 10.16 | 62.81 ± 10.99 | 63.98 ± 11.69 | <.001 |
| Heart rate, bpm (mean) | SOFA Cardiovascular (baseline) | 87.86 ± 16.39 | 87.75 ± 15.93 | 88.40 ± 16.27 | .24 |
| Dopamine, µg/kg/min (mean) | SOFA Cardiovascular (vasopressors) | 7.82 ± 4.48 | 6.45 ± 3.81 | 5.77 ± 3.00 | .03 |
| Norepinephrine, µg/kg/min (mean) | SOFA Cardiovascular (vasopressors) | 0.15 ± 0.12 | 0.14 ± 0.13 | 0.10 ± 0.09 | <.001 |
| Dobutamine, µg/kg/min (mean) | SOFA Cardiovascular (vasopressors) | 4.51 ± 2.81 | 3.72 ± 1.97 | 4.65 ± 3.18 | .26 |
| Epinephrine, µg/kg/min (mean) | SOFA Cardiovascular (vasopressors) | 0.08 ± 0.13 | 0.07 ± 0.08 | 0.14 ± 0.14 | .16 |
| Other vasopressor/inotrope, any | SOFA Cardiovascular (vasopressors) | 5,567 (27.0%) | 402 (14.8%) | 114 (9.8%) | <.001 |
| Mechanical circulatory support, any | SOFA Cardiovascular (mechanical support) | 459 (2.2%) | 33 (1.2%) | 15 (1.3%) | <.001 |
| Glasgow Coma Scale score (mean) | SOFA CNS (baseline) | 14.26 ± 1.40 | 14.59 ± 1.15 | 14.52 ± 1.13 | <.001 |
| Delirium treatment, any | SOFA CNS (updated) | 2,169 (10.5%) | 314 (11.5%) | 124 (10.7%) | .27 |
| GCS motor score (mean) | SOFA CNS (updated) | 4.90 ± 1.48 | 5.39 ± 1.17 | 5.41 ± 1.21 | <.001 |
| Creatinine, mg/dL (mean) | SOFA Renal (baseline) | 1.72 ± 1.62 | 1.28 ± 1.26 | 1.46 ± 1.30 | <.001 |
|  |  |  |  |  |  |
| *(continue)* | | | | | |
| **Characteristic** | **Category** | **Concordant Positive** | **SOFA-2 Only** | **SOFA-1 Only** | **P value** |
| 24-h urine output, mL (mean) | SOFA Renal (baseline) | 1,809.66 ± 1,345.94 | 1,946.06 ± 1,198.68 | 1,899.77 ± 1,618.79 | <.001 |
| 6-h urine output, mL/kg/h (mean) | SOFA Renal (updated) | 1.58 ± 4.23 | 1.77 ± 1.92 | 2.37 ± 4.53 | <.001 |
| 12-h urine output, mL/kg/h (mean) | SOFA Renal (updated) | 1.33 ± 3.06 | 1.47 ± 1.66 | 1.99 ± 4.67 | <.001 |
| 24-h urine output, mL/kg/h (mean) | SOFA Renal (updated) | 0.99 ± 2.24 | 1.10 ± 1.39 | 1.58 ± 3.97 | <.001 |
| Met criteria for RRT, any | SOFA Renal (updated) | 1,056 (5.1%) | 58 (2.1%) | 10 (0.9%) | <.001 |
| Potassium, mmol/L (mean) | SOFA Renal (updated) | 4.18 ± 0.58 | 4.04 ± 0.52 | 4.09 ± 0.53 | <.001 |
| Bicarbonate, mmol/L (mean) | SOFA Renal (updated) | 22.63 ± 4.87 | 24.19 ± 4.64 | 23.66 ± 4.80 | <.001 |
| Respiratory rate, breaths/min (mean) | Others | 20.10 ± 4.32 | 20.21 ± 4.42 | 20.49 ± 4.20 | .004 |
| Temperature, °C (mean) | Others | 36.96 ± 0.60 | 37.00 ± 0.53 | 36.95 ± 0.53 | .002 |
| Weight, kg (mean) | Others | 82.47 ± 24.63 | 83.04 ± 26.54 | 79.39 ± 24.38 | <.001 |
| White blood cell count (mean) | Others | 13.62 ± 10.58 | 12.81 ± 8.88 | 13.09 ± 7.60 | <.001 |
| Lactate, mmol/L (mean) | Others | 2.32 ± 1.88 | 1.80 ± 1.28 | 1.82 ± 1.32 | <.001 |
| PaCO_2_, mm Hg (mean) | Others | 41.82 ± 10.10 | 42.29 ± 10.66 | 42.83 ± 11.16 | .07 |
| Arterial pH (mean) | Others | 7.36 ± 0.07 | 7.39 ± 0.07 | 7.38 ± 0.07 | <.001 |
| Urine output, mL/h (mean) | Others | 130.88 ± 122.81 | 156.85 ± 139.43 | 201.39 ± 164.81 | <.001 |

eTable 4. Detailed Baseline Characteristics by Diagnostic Group (eICU-CRD)

| **Characteristic** | **Category** | **Concordant Positive** | **SOFA-2 Only** | **SOFA-1 Only** | **P value** |
| --- | --- | --- | --- | --- | --- |
| No. of patients | Sample size | 8,841 | 2,006 | 1,362 |  |
| Age, y | Demographics | 64.69 ± 15.79 | 65.07 ± 15.96 | 66.04 ± 16.13 | .004 |
| Male sex | Demographics | 4,861 (55.0%) | 1,057 (52.7%) | 695 (51.0%) | .008 |
| ICU length of stay, d (median) | Demographics | 4.10 (2.33–8.06) | 3.32 (1.94–6.22) | 3.27 (1.96–5.90) | <.001 |
| PaO_2_, mm Hg (mean) | SOFA-1 Respiratory | 111.49 ± 52.35 | 103.25 ± 45.17 | 103.42 ± 46.65 | <.001 |
| FiO_2_, % (mean) | SOFA-1 Respiratory | 51.89 ± 18.99 | 42.55 ± 17.29 | 45.98 ± 19.06 | <.001 |
| PaO_2_/FiO_2_ ratio (mean) | SOFA-1 Respiratory | 245.89 ± 145.44 | 251.89 ± 134.45 | 266.82 ± 136.12 | <.001 |
| Mechanical ventilation, any | SOFA-1 Respiratory | 6,066 (68.6%) | 1,379 (68.7%) | 759 (55.7%) | <.001 |
| SpO_2_, % (mean) | SOFA-2 Respiratory (updated) | 96.71 ± 2.63 | 96.30 ± 2.29 | 96.77 ± 2.22 | <.001 |
| SpO_2_/FiO_2_ ratio (mean) | SOFA-2 Respiratory (updated) | 218.20 ± 78.13 | 266.55 ± 94.32 | 250.41 ± 90.37 | <.001 |
| Advanced respiratory support, any | SOFA-2 Respiratory (updated) | 5,189 (58.7%) | 991 (49.4%) | 440 (32.3%) | <.001 |
| Platelets, ×10³/µL (mean) | SOFA-2 Coagulation | 188.09 ± 107.17 | 217.67 ± 109.08 | 220.49 ± 114.48 | <.001 |
| Total bilirubin, mg/dL (mean) | SOFA Liver | 1.79 ± 3.41 | 1.21 ± 2.66 | 1.19 ± 2.56 | <.001 |
| Mean arterial pressure, mm Hg (mean) | SOFA Cardiovascular (baseline) | 76.63 ± 11.03 | 80.29 ± 12.63 | 79.36 ± 12.14 | <.001 |
| Systolic blood pressure, mm Hg (mean) | SOFA Cardiovascular (baseline) | 112.38 ± 17.72 | 116.96 ± 19.87 | 117.57 ± 18.78 | <.001 |
| Diastolic blood pressure, mm Hg (mean) | SOFA Cardiovascular (baseline) | 56.39 ± 10.22 | 57.52 ± 11.21 | 58.04 ± 11.69 | .02 |
| Heart rate, bpm (mean) | SOFA Cardiovascular (baseline) | 90.68 ± 17.27 | 88.64 ± 16.20 | 87.94 ± 16.45 | <.001 |
| Dopamine, µg/kg/min (mean) | SOFA Cardiovascular (vasopressors) | 8.00 ± 7.83 | 7.15 ± 6.07 | 6.75 ± 6.67 | .59 |
| Norepinephrine, µg/kg/min (mean) | SOFA Cardiovascular (vasopressors) | 0.21 ± 0.23 | 0.20 ± 0.22 | 0.10 ± 0.13 | <.001 |
| Dobutamine, µg/kg/min (mean) | SOFA Cardiovascular (vasopressors) | 5.91 ± 4.42 | 6.09 ± 3.73 | 3.93 ± 3.05 | .33 |
| Epinephrine, µg/kg/min (mean) | SOFA Cardiovascular (vasopressors) | 0.18 ± 0.20 | 0.07 ± 0.05 | 0.05 ± 0.07 | .04 |
| Other vasopressor/inotrope, any | SOFA Cardiovascular (vasopressors) | 1,431 (16.2%) | 168 (8.4%) | 65 (4.8%) | <.001 |
| Mechanical circulatory support, any | SOFA Cardiovascular (mechanical support) | 39 (0.4%) | 8 (0.4%) | 3 (0.2%) | .49 |
| Glasgow Coma Scale score (mean) | SOFA CNS (baseline) | 11.95 ± 3.15 | 13.19 ± 2.71 | 13.38 ± 2.46 | <.001 |
| Delirium treatment, any | SOFA CNS (updated) | 260 (2.9%) | 52 (2.6%) | 24 (1.8%) | .04 |
| GCS motor score (mean) | SOFA CNS (updated) | 5.27 ± 1.17 | 5.63 ± 0.88 | 5.68 ± 0.78 | <.001 |
| Creatinine, mg/dL (mean) | SOFA Renal (baseline) | 1.91 ± 1.64 | 1.47 ± 1.50 | 1.60 ± 1.45 | <.001 |
| 24-h urine output, mL (mean) | SOFA Renal (baseline) | 1,590.13 ± 1,269.92 | 1,638.23 ± 1,183.00 | 1,534.90 ± 1,195.42 | .58 |
|  |  |  |  |  |  |
| *(continue)* | | | | | |
| **Characteristic** | **Category** | **Concordant Positive** | **SOFA-2 Only** | **SOFA-1 Only** | **P value** |
| 6-h urine output, mL/kg/h (mean) | SOFA Renal (updated) | 3.42 ± 3.43 | 3.72 ± 3.42 | 4.00 ± 3.40 | <.001 |
| 12-h urine output, mL/kg/h (mean) | SOFA Renal (updated) | 3.06 ± 2.83 | 3.41 ± 3.14 | 3.61 ± 2.92 | <.001 |
| 24-h urine output, mL/kg/h (mean) | SOFA Renal (updated) | 2.67 ± 2.31 | 3.06 ± 2.80 | 3.09 ± 2.21 | .002 |
| Renal replacement therapy, any | SOFA Renal (updated) | 553 (6.3%) | 89 (4.4%) | 33 (2.4%) | <.001 |
| Met criteria for RRT, any | SOFA Renal (updated) | 315 (3.6%) | 36 (1.8%) | 13 (1.0%) | <.001 |
| Potassium, mmol/L (mean) | SOFA Renal (updated) | 4.08 ± 0.63 | 3.98 ± 0.58 | 4.00 ± 0.57 | <.001 |
| Bicarbonate, mmol/L (mean) | SOFA Renal (updated) | 22.74 ± 5.31 | 24.74 ± 5.25 | 23.57 ± 5.14 | <.001 |
| Respiratory rate, breaths/min (mean) | Others | 20.88 ± 5.57 | 20.97 ± 5.62 | 21.14 ± 5.46 | .38 |
| Temperature, °C (mean) | Others | 37.11 ± 1.19 | 37.37 ± 1.08 | 37.11 ± 0.82 | .007 |
| Weight, kg (mean) | Others | 85.45 ± 28.32 | 86.51 ± 29.98 | 81.96 ± 26.53 | <.001 |
| White blood cell count (mean) | Others | 14.22 ± 9.46 | 13.23 ± 7.21 | 13.66 ± 8.39 | .21 |
| Lactate, mmol/L (mean) | Others | 2.76 ± 2.74 | 2.04 ± 2.03 | 2.01 ± 1.64 | <.001 |
| PaCO_2_, mm Hg (mean) | Others | 40.67 ± 11.54 | 42.00 ± 12.19 | 41.23 ± 12.09 | .07 |
| Arterial pH (mean) | Others | 7.35 ± 0.09 | 7.38 ± 0.08 | 7.37 ± 0.08 | <.001 |
| Urine output, mL/h (mean) | Others | 305.69 ± 320.31 | 324.45 ± 295.16 | 372.17 ± 341.38 | <.001 |

eTable 5. Detailed Baseline Characteristics by Diagnostic Group (AmsterdamUMCdb)

| Characteristic | Category | Concordant Positive | SOFA-2 Only | SOFA-1 Only | P value |
| --- | --- | --- | --- | --- | --- |
| No. of patients | Sample size | 4,225 | 373 | 122 |  |
| Age, y | Demographics | 61.61 ± 16.39 | 62.83 ± 15.75 | 58.98 ± 18.18 | .19 |
| Male sex | Demographics | 2,703 (64.9%) | 221 (60.5%) | 78 (64.5%) | .25 |
| ICU length of stay, d (median) | Demographics | 6.67 (3.12–14.25) | 5.67 (2.92–11.54) | 4.65 (2.80–10.70) | .012 |
| PaO_2_, mm Hg (mean) | SOFA-1 Respiratory | 104.69 ± 27.96 | 100.21 ± 23.87 | 104.27 ± 27.34 | .01 |
| FiO_2_, % (mean) | SOFA-1 Respiratory | 47.57 ± 11.45 | 44.71 ± 9.19 | 45.18 ± 9.02 | <.001 |
| PaO_2_/FiO_2_ ratio (mean) | SOFA-1 Respiratory | 270.33 ± 113.70 | 276.22 ± 111.82 | 334.71 ± 125.46 | <.001 |
| Mechanical ventilation, any | SOFA-1 Respiratory | 3,871 (91.6%) | 321 (86.1%) | 85 (69.7%) | <.001 |
| SpO_2_, % (mean) | SOFA-2 Respiratory (updated) | 97.36 ± 2.13 | 97.02 ± 2.13 | 97.58 ± 1.80 | <.001 |
| SpO_2_/FiO_2_ ratio (mean) | SOFA-2 Respiratory (updated) | 216.60 ± 49.92 | 225.91 ± 45.29 | 225.16 ± 42.20 | <.001 |
| Advanced respiratory support, any | SOFA-2 Respiratory (updated) | 3,895 (92.2%) | 325 (87.1%) | 83 (68.0%) | <.001 |
| ECMO, any | SOFA-2 Respiratory (updated) | 2 (0.0%) | 0 (0.0%) | 0 (0.0%) | .89 |
| Platelets, ×10³/µL (mean) | SOFA-2 Coagulation | 193.51 ± 108.72 | 223.80 ± 113.50 | 207.79 ± 106.60 | <.001 |
| Total bilirubin, mg/dL (mean) | SOFA Liver | 1.07 ± 1.78 | 0.81 ± 1.67 | 1.03 ± 1.74 | <.001 |
| Mean arterial pressure, mm Hg (mean) | SOFA Cardiovascular (baseline) | 79.39 ± 9.94 | 81.50 ± 11.09 | 81.94 ± 10.00 | <.001 |
| Systolic blood pressure, mm Hg (mean) | SOFA Cardiovascular (baseline) | 118.60 ± 18.02 | 123.44 ± 19.67 | 122.11 ± 18.31 | <.001 |
| Diastolic blood pressure, mm Hg (mean) | SOFA Cardiovascular (baseline) | 59.85 ± 8.59 | 60.66 ± 8.90 | 61.46 ± 8.42 | .06 |
| Heart rate, bpm (mean) | SOFA Cardiovascular (baseline) | 83.17 ± 20.19 | 84.11 ± 17.83 | 85.23 ± 18.68 | .23 |
| Dopamine, µg/kg/min (mean) | SOFA Cardiovascular (vasopressors) | 3.86 ± 2.45 | 3.52 ± 1.94 | 4.45 ± 2.54 | .57 |
| Norepinephrine, µg/kg/min (mean) | SOFA Cardiovascular (vasopressors) | 0.25 ± 0.27 | 0.27 ± 0.25 | 0.18 ± 0.25 | <.001 |
| Dobutamine, µg/kg/min (mean) | SOFA Cardiovascular (vasopressors) | 4.06 ± 2.50 | 4.13 ± 2.57 | 3.59 ± 1.59 | .97 |
| Epinephrine, µg/kg/min (mean) | SOFA Cardiovascular (vasopressors) | 0.29 ± 0.25 | – | – | .24 |
| Other vasopressor/inotrope, any | SOFA Cardiovascular (vasopressors) | 776 (18.4%) | 44 (11.8%) | 5 (4.1%) | <.001 |
| Mechanical circulatory support, any | SOFA Cardiovascular (mechanical support) | 85 (2.0%) | 2 (0.5%) | 1 (0.8%) | .09 |
| Glasgow Coma Scale score (mean) | SOFA CNS (baseline) | 13.26 ± 3.31 | 14.60 ± 1.68 | 14.47 ± 1.64 | <.001 |
| Delirium treatment, any | SOFA CNS (updated) | 273 (6.5%) | 32 (8.6%) | 12 (9.8%) | .11 |
| GCS motor score (mean) | SOFA CNS (updated) | 4.24 ± 2.01 | 4.88 ± 1.75 | 4.89 ± 1.70 | <.001 |
| Creatinine, mg/dL (mean) | SOFA Renal (baseline) | 1.42 ± 1.21 | 1.13 ± 0.84 | 1.26 ± 1.15 | <.001 |
| 24-h urine output, mL (mean) | SOFA Renal (baseline) | 1,961.01 ± 1,479.07 | 1,830.94 ± 1,161.70 | 2,653.68 ± 1,484.86 | <.001 |
|  |  |  |  |  |  |
| *(continue)* | | | | | |
| Characteristic | Category | Concordant Positive | SOFA-2 Only | SOFA-1 Only | P value |
| 6-h urine output, mL/kg/h (mean) | SOFA Renal (updated) | 1.23 ± 0.94 | 1.21 ± 0.79 | 2.01 ± 1.16 | <.001 |
| 12-h urine output, mL/kg/h (mean) | SOFA Renal (updated) | 1.16 ± 0.90 | 1.13 ± 0.74 | 1.91 ± 1.11 | <.001 |
| 24-h urine output, mL/kg/h (mean) | SOFA Renal (updated) | 0.93 ± 0.70 | 0.96 ± 0.62 | 1.54 ± 0.83 | <.001 |
| Met criteria for RRT, any | SOFA Renal (updated) | 209 (4.9%) | 6 (1.6%) | 3 (2.5%) | .007 |
| Potassium, mmol/L (mean) | SOFA Renal (updated) | 4.15 ± 0.45 | 4.08 ± 0.37 | 4.03 ± 0.40 | .006 |
| Bicarbonate, mmol/L (mean) | SOFA Renal (updated) | 21.84 ± 4.21 | 23.66 ± 4.65 | 23.09 ± 4.04 | <.001 |
| Respiratory rate, breaths/min (mean) | Others | 19.42 ± 4.64 | 19.57 ± 4.85 | 18.68 ± 4.35 | .30 |
| Temperature, °C (mean) | Others | 35.18 ± 1.96 | 35.52 ± 1.96 | 36.03 ± 1.84 | .008 |
| Weight, kg (mean) | Others | 80.12 ± 15.93 | 79.74 ± 17.14 | 75.57 ± 16.50 | .02 |
| White blood cell count (mean) | Others | 13.59 ± 8.97 | 14.06 ± 7.77 | 13.48 ± 6.92 | .57 |
| Lactate, mmol/L (mean) | Others | 2.76 ± 2.46 | 1.96 ± 1.61 | 2.15 ± 2.09 | <.001 |
| PaCO_2_, mm Hg (mean) | Others | 40.38 ± 7.38 | 42.07 ± 9.28 | 39.77 ± 8.06 | <.001 |
| Arterial pH (mean) | Others | 7.35 ± 0.07 | 7.37 ± 0.06 | 7.38 ± 0.07 | <.001 |
| Urine output, mL/h (mean) | Others | 96.92 ± 69.10 | 99.11 ± 63.42 | 146.53 ± 83.44 | <.001 |

eTable 6. Clinical Severity and Organ Support Utilization Among Sepsis-Positive Patients

| **Metric** | **Overall** | **Concordant Positivea** | **SOFA-2 Onlya** | **SOFA-1 Onlyb** | **P** **value** |
| --- | --- | --- | --- | --- | --- |
| ***MIMIC-IV (N = 24,521)*** |  |  |  |  |  |
| No. of patients (%) | 24,521 (100.0%) | 20,635 (84.2%) | 2,724 (11.1%) | 1,162 (4.7%) | *<*.001 |
| Total SOFA score at diagnosis, mean *±* SD | 5.4 ± 2.9 | 5.4 *±* 2.9 | 5.5 *±* 2.8 | 5.3 *±* 2.8 | *<*.001 |
| **Organ Support Utilization, n (%)** |  |  |  |  |  |
| Invasive mechanical ventilation | 12,870 (52.5%) | 11,442 (55.4%) | 1,052 (38.6%) | 376 (32.4%) | *<*.001 |
| HFNC or noninvasive ventilation | 15,968 (65.1%) | 13,903 (67.4%) | 1,565 (57.5%) | 500 (43.0%) | *<*.001 |
| Vasopressors | 10,516 (42.9%) | 9,570 (46.4%) | 649 (23.8%) | 297 (25.6%) | *<*.001 |
| Renal replacement therapy | 1,124 (4.6%) | 1,056 (5.1%) | 58 (2.1%) | 10 (0.9%) | *<*.001 |
| Mechanical circulatory support^c^ | 507 (2.1%) | 459 (2.2%) | 33 (1.2%) | 15 (1.3%) | *<*.001 |
| ***eICU-CRD (N = 12,209)*** |  |  |  |  |  |
| No. of patients (%) | 12,209 (100.0%) | 8,841 (72.4%) | 2,006 (16.4%) | 1,362 (11.2%) | *<*.001 |
| Total SOFA score at diagnosis, mean *±* SD | 6.0 ± 2.8 | 6.0 *±* 2.8 | 5.9 *±* 2.8 | 6.0 *±* 2.6 | *<*.001 |
| **Organ Support Utilization, n (%)** |  |  |  |  |  |
| Invasive mechanical ventilation | 8,204 (67.2%) | 6,066 (68.6%) | 1,379 (68.7%) | 759 (55.7%) | *<*.001 |
| HFNC or noninvasive ventilation | 6,620 (54.2%) | 5,189 (58.7%) | 991 (49.4%) | 440 (32.3%) | *<*.001 |
| Vasopressors | 4,263 (34.9%) | 3,619 (40.9%) | 298 (14.9%) | 346 (25.4%) | *<*.001 |
| Renal replacement therapy | 999 (8.2%) | 833 (9.4%) | 121 (6.0%) | 45 (3.3%) | *<*.001 |
| Mechanical circulatory support^c^ | 50 (0.4%) | 39 (0.4%) | 8 (0.4%) | 3 (0.2%) | .49 |
| ***AmsterdamUMCdb (N = 4,720)*** |  |  |  |  |  |
| No. of patients (%) | 4,720 (100.0%) | 4,225 (89.5%) | 373 (7.9%) | 122 (2.6%) | *<*.001 |
| Total SOFA score at diagnosis, mean *±* SD | 5.9 ± 2.8 | 5.8 *±* 2.8 | 6.8 *±* 2.6 | 6.1 *±* 3.3 | *<*.001 |
| **Organ Support Utilization, n (%)** |  |  |  |  |  |
| Invasive mechanical ventilation | 4,277 (90.6%) | 3,871 (91.6%) | 321 (86.1%) | 85 (69.7%) | *<*.001 |
| HFNC or noninvasive ventilation | 4,303 (91.2%) | 3,895 (92.2%) | 325 (87.1%) | 83 (68.0%) | *<*.001 |
| Vasopressors | 3,843 (81.4%) | 3,544 (83.9%) | 227 (60.9%) | 72 (59.0%) | *<*.001 |
| Renal replacement therapy | 218 (4.6%) | 209 (4.9%) | 6 (1.6%) | 3 (2.5%) | .007 |
| Mechanical circulatory support^c^ | 88 (1.9%) | 85 (2.0%) | 2 (0.5%) | 1 (0.8%) | .09 |

Total SOFA scores were assessed at the time of sepsis diagnosis, whereas organ-support utilization was defined as the proportion of patients receiving organ-support within the first 24 hours after sepsis diagnosis.

Abbreviations: HFNC, high-flow nasal cannula; SOFA, Sequential Organ Failure Assessment.

^a^ Scores for the Concordant Positive and SOFA-2 Only groups are calculated using the SOFA-2 criteria.

^b^ Scores for the SOFA-1 Only group are calculated using the SOFA-1 criteria.

^c^ Includes intra-aortic balloon pump and extracorporeal membrane oxygenation.

eTable 7. SOFA-2 Only Group: Clinical Characteristics at Sepsis Diagnosis

| **Characteristic** | **MIMIC-IV (n=2724)** | **eICU-CRD (n=2006)** | **Amsterdam UMC (n=373)** | |
| --- | --- | --- | --- | --- |
| **Respiratory System** |  |  |  | |
| PaO_2_/FiO_2_ ratio | 330.01 ± 195.84 | 251.89 ± 134.45 | 276.22 ± 111.82 | |
| P/F < 400 (SOFA ≥1) | 735/1061 (69.3%) | 530/608 (87.2%) | 318/370 (85.9%) | |
| P/F < 300 (SOFA ≥2) | 544/1061 (51.3%) | 438/608 (72.0%) | 240/370 (64.9%) | |
| P/F ≤ 200 (SOFA ≥3) | 290/1061 (27.3%) | 236/608 (38.8%) | 98/370 (26.5%) | |
| P/F ≤ 100 (SOFA = 4) | 62/1061 (5.8%) | 36/608 (5.9%) | 7/370 (1.9%) | |
| SpO_2_/FiO_2_ ratio (SpO_2_ < 98%) | 205.80 ± 56.06 | 269.00 ± 98.88 | 217.58 ± 47.99 | |
| S/F < 400 (SOFA ≥1) | 1342/1347 (99.6%) | 1021/1204 (84.8%) | 201/201 (100.0%) | |
| S/F < 315 (SOFA ≥2) | 1290/1347 (95.8%) | 858/1204 (71.3%) | 191/201 (95.0%) | |
| S/F < 235 (SOFA ≥3) | 869/1347 (64.5%) | 483/1204 (40.1%) | 138/201 (68.7%) | |
| S/F < 150 (SOFA = 4) | 260/1347 (19.3%) | 100/1204 (8.3%) | 12/201 (6.0%) | |
| SpO_2_ (%) | 96.13 ± 2.03 | 96.30 ± 2.29 | 97.02 ± 2.13 | |
| SpO_2_ < 98% (S/F applicable) | 2182/2720 (80.2%) | 1535/1994 (77.0%) | 245/373 (65.7%) | |
| SpO_2_ < 98% & P/F unavailable | 1402/2720 (51.5%) | 1120/1994 (56.2%) | 3/373 (0.8%) | |
| FiO_2_ (%) | 49.84 ± 16.07 | 42.55 ± 17.29 | 44.71 ± 9.19 | |
| HFNC/NIV use | 1565 (57.45%) | 991 (49.40%) | 325 (87.13%) | |
| Invasive MV | 1052 (38.62%) | 1379 (68.74%) | 321 (86.06%) | |
| ECMO use | 8 (0.29%) | N/A | 0 (0.00%) | |
| Any advanced respiratory support | 1590 (58.37%) | 1439 (71.73%) | 327 (87.67%) | |
| SOFA-1 respiratory score | 1.47 ± 1.26 | 1.32 ± 1.40 | 2.59 ± 1.11 | |
| SOFA-2 respiratory score | 2.06 ± 1.72 | 2.21 ± 1.47 | 2.68 ± 1.44 | |
| Respiratory score difference | 1.17 ± 1.63 | 1.23 ± 1.46 | 0.12 ± 0.98 | |
| **Renal System** |  |  |  | |
| Serum creatinine (mg/dL) | 1.28 ± 1.26 | 1.47 ± 1.50 | 1.13 ± 0.84 | |
| Cr ≥ 1.2 (SOFA ≥1) | 812/2664 (30.5%) | 686/1890 (36.3%) | 94/359 (26.2%) | |
| Cr ≥ 2.0 (SOFA ≥2) | 359/2664 (13.5%) | 317/1890 (16.8%) | 28/359 (7.8%) | |
| Cr ≥ 3.5 (SOFA ≥3) | 146/2664 (5.5%) | 158/1890 (8.4%) | 10/359 (2.8%) | |
| Cr ≥ 5.0 (SOFA = 4) | 68/2664 (2.6%) | 82/1890 (4.3%) | 5/359 (1.4%) | |
| Body weight (kg) | 83.04 ± 26.54 | 86.51 ± 29.98 | 79.74 ± 17.14 | |
| 24h absolute urine output (mL/day) | 1946.06 ± 1198.68 | 1638.23 ± 1183.00 | 1830.94 ± 1161.70 | |
| UO < 500 mL/day (SOFA-1 ≥3) | 55/2589 (2.1%) | 51/257 (19.8%) | 3/367 (0.8%) | |
| UO < 200 mL/day (SOFA-1 = 4) | 26/2589 (1.0%) | 31/257 (12.1%) | 2/367 (0.5%) | |
| 24h normalized urine output (mL/kg/h) | 1.10 ± 1.39 | 3.06 ± 2.80 | 0.96 ± 0.62 | |
| 12h normalized urine output (mL/kg/h) | 1.47 ± 1.66 | 3.41 ± 3.14 | 1.13 ± 0.74 | |
| 6h normalized urine output (mL/kg/h) | 1.77 ± 1.92 | 3.72 ± 3.42 | 1.21 ± 0.79 | |
| Minimum urine output window (mL/kg/h) | 1.31 ± 1.52 | 3.01 ± 2.72 | 0.92 ± 0.61 | |
| UO < 0.5 mL/kg/h (SOFA-2 ≥3) | 422/2403 (17.6%) | 23/257 (8.9%) | 68/359 (18.9%) | |
| UO < 0.3 mL/kg/h (SOFA-2 = 4) | 101/2403 (4.2%) | 15/257 (5.8%) | 8/359 (2.2%) | |
| RRT use | 58 (2.13%) | 36 (1.79%) | 6 (1.61%) | |
| SOFA-1 renal score | 0.62 ± 1.04 | 0.91 ± 1.28 | 0.43 ± 0.81 | |
| SOFA-2 renal score | 1.00 ± 1.11 | 1.23 ± 1.25 | 1.29 ± 1.04 | |
| Renal score difference | 0.42 ± 0.84 | 0.32 ± 1.01 | 0.86 ± 0.91 | |
| **Cardiovascular System** |  |  |  | |
| Mean arterial pressure (mm Hg) | 77.86 ± 10.80 | 80.29 ± 12.63 | 81.50 ± 11.09 | |
| MAP < 70 mm Hg (SOFA ≥1) | 640/2718 (23.5%) | 428/1999 (21.4%) | 44/371 (11.9%) | |
| Lactate (mmol/L) | 1.80 ± 1.28 | 2.04 ± 2.03 | 1.96 ± 1.61 | |
| Lactate > 2 (SOFA-2 ≥2) | 293/1106 (26.5%) | 168/551 (30.5%) | 62/213 (29.1%) | |
| Lactate > 4 (SOFA-2 ≥3) | 41/1106 (3.7%) | 38/551 (6.9%) | 18/213 (8.5%) | |
|  | | | |  |
| Lactate > 8 (SOFA-2 = 4) | 8/1106 (0.7%) | 10/551 (1.8%) | 4/213 (1.9%) | |
|  |  |  |  | |
| Dopamine use | 22 (0.81%) | 15 (0.75%) | 28 (7.51%) | |
| Dopamine > 5 μg/kg/min (SOFA ≥3) | 13/22 (59.1%) | 8/16 (50.0%) | 8/28 (28.6%) | |
| Dopamine > 15 μg/kg/min (SOFA = 4) | 1/22 (4.5%) | 2/16 (12.5%) | 0/28 (0.0%) | |
| Norepinephrine use | 330 (12.11%) | 185 (9.22%) | 209 (56.03%) | |
| Norepinephrine > 0.1 μg/kg/min | 163/330 (49.4%) | 99/194 (51.0%) | 158/209 (75.6%) | |
|  |  |  |  | |
| *(continue)* | | | |  |
| **Characteristic** | **MIMIC-IV (n=2724)** | **eICU-CRD (n=2006)** | **Amsterdam UMC (n=373)** | |
| Epinephrine use | 25 (0.92%) | 10 (0.50%) | 1 (0.27%) | |
| Epinephrine > 0.1 μg/kg/min (SOFA = 4) | 7/25 (28.0%) | 2/10 (20.0%) | 1/1 (100.0%) | |
| Dobutamine use | 26 (0.95%) | 7 (0.35%) | 25 (6.70%) | |
| Any vasopressor use | 364 (13.36%) | 199 (9.92%) | 223 (59.79%) | |
| SOFA-1 cardiovascular score | 1.12 ± 1.17 | 1.02 ± 1.02 | 2.61 ± 1.59 | |
| SOFA-2 cardiovascular score | 1.14 ± 1.00 | 1.02 ± 0.91 | 2.16 ± 1.35 | |
| Cardiovascular score difference | 0.02 ± 0.65 | 0.00 ± 0.45 | −0.45 ± 0.72 | |
| **Overall SOFA Score** |  |  |  | |
| Total SOFA-1 score | 3.77 ± 3.02 | 4.39 ± 3.22 | 6.24 ± 2.78 | |
| Total SOFA-2 score | 5.52 ± 2.85 | 5.86 ± 2.78 | 6.84 ± 2.65 | |

Abbreviations: Cr, creatinine; ECMO, extracorporeal membrane oxygenation; HFNC, high-flow nasal cannula; MAP, mean arterial pressure; MV, mechanical ventilation; NIV, noninvasive ventilation; P/F, PaO_2_/FiO_2_; RRT, renal replacement therapy; S/F, SpO_2_/FiO_2_; SOFA, Sequential Organ Failure Assessment; UO, urine output.

Note: Continuous variables presented as mean ± SD; categorical variables as n (%). Score difference = SOFA-2 − SOFA-1. SpO_2_/FiO_2_ calculated only when SpO_2_ < 98% (SOFA-2 criterion). Threshold statistics: n/N (x%) indicates n patients meeting criterion among N with available data.

eTable 8. SOFA-1 Only Group: Clinical Characteristics at Sepsis Diagnosis

| **Characteristic** | **MIMIC-IV (n=1162)** | **eICU-CRD (n=1362)** | **Amsterdam UMC (n=122)** | |
| --- | --- | --- | --- | --- |
| **Respiratory System** |  |  |  | |
| PaO_2_/FiO_2_ ratio | 281.89 ± 169.65 | 266.82 ± 136.12 | 334.71 ± 125.46 | |
| P/F < 400 (SOFA ≥1) | 596/712 (83.7%) | 595/673 (88.4%) | 87/120 (72.5%) | |
| P/F < 300 (SOFA ≥2) | 450/712 (63.2%) | 429/673 (63.7%) | 50/120 (41.7%) | |
| P/F ≤ 200 (SOFA ≥3) | 252/712 (35.4%) | 238/673 (35.4%) | 18/120 (15.0%) | |
| P/F ≤ 100 (SOFA = 4) | 41/712 (5.8%) | 61/673 (9.1%) | 0/120 (0.0%) | |
| SpO_2_/FiO_2_ ratio (SpO_2_ < 98%) | 201.95 ± 59.38 | 238.93 ± 96.08 | 205.79 ± 46.94 | |
| S/F < 400 (SOFA ≥1) | 374/380 (98.4%) | 391/428 (91.4%) | 30/30 (100.0%) | |
| S/F < 315 (SOFA ≥2) | 361/380 (95.0%) | 340/428 (79.4%) | 28/30 (93.3%) | |
| S/F < 235 (SOFA ≥3) | 283/380 (74.5%) | 226/428 (52.8%) | 24/30 (80.0%) | |
| S/F < 150 (SOFA = 4) | 71/380 (18.7%) | 78/428 (18.2%) | 4/30 (13.3%) | |
| SpO_2_ (%) | 95.99 ± 2.02 | 96.77 ± 2.22 | 97.58 ± 1.80 | |
| SpO_2_ < 98% (S/F applicable) | 936/1150 (81.4%) | 897/1344 (66.7%) | 62/120 (51.7%) | |
| SpO_2_ < 98% & P/F unavailable | 401/1150(34.9%) | 472/1344(35.1%) | 1/120(0.8%) | |
| FiO_2_ (%) | 50.00 ± 14.31 | 45.98 ± 19.06 | 45.18 ± 9.02 | |
| HFNC/NIV use | 500 (43.03%) | 440 (32.31%) | 83 (68.03%) | |
| Invasive MV | 376 (32.36%) | 759 (55.73%) | 85 (69.67%) | |
| ECMO use | 1 (0.09%) | N/A | 0 (0.00%) | |
| Any advanced respiratory support | 514 (44.23%) | 785 (57.64%) | 85 (69.67%) | |
| SOFA-1 respiratory score | 1.85 ± 1.10 | 1.62 ± 1.29 | 2.07 ± 1.26 | |
| SOFA-2 respiratory score | 1.72 ± 1.82 | 1.21 ± 1.42 | 1.59 ± 1.63 | |
| Respiratory score difference | 0.40 ± 1.36 | −0.11 ± 1.13 | −0.50 ± 0.85 | |
| **Renal System** |  |  |  | |
| Serum creatinine (mg/dL) | 1.46 ± 1.30 | 1.60 ± 1.45 | 1.26 ± 1.15 | |
| Cr ≥ 1.2 (SOFA ≥1) | 438/1130 (38.8%) | 570/1287 (44.3%) | 33/121 (27.3%) | |
| Cr ≥ 2.0 (SOFA ≥2) | 236/1130 (20.9%) | 288/1287 (22.4%) | 17/121 (14.0%) | |
| Cr ≥ 3.5 (SOFA ≥3) | 102/1130 (9.0%) | 131/1287 (10.2%) | 7/121 (5.8%) | |
| Cr ≥ 5.0 (SOFA = 4) | 33/1130 (2.9%) | 55/1287 (4.3%) | 3/121 (2.5%) | |
| Body weight (kg) | 79.39 ± 24.38 | 81.96 ± 26.53 | 75.57 ± 16.50 | |
| 24h absolute urine output (mL/day) | 1899.77 ± 1618.79 | 1534.90 ± 1195.42 | 2653.68 ± 1484.86 | |
| UO < 500 mL/day (SOFA-1 ≥3) | 173/1077 (16.1%) | 40/166 (24.1%) | 9/118 (7.6%) | |
| UO < 200 mL/day (SOFA-1 = 4) | 72/1077 (6.7%) | 27/166 (16.3%) | 4/118 (3.4%) | |
| 24h normalized urine output (mL/kg/h) | 1.58 ± 3.97 | 3.09 ± 2.21 | 1.54 ± 0.83 | |
| 12h normalized urine output (mL/kg/h) | 1.99 ± 4.67 | 3.61 ± 2.92 | 1.91 ± 1.11 | |
| 6h normalized urine output (mL/kg/h) | 2.37 ± 4.53 | 4.00 ± 3.40 | 2.01 ± 1.16 | |
| Minimum urine output window (mL/kg/h) | 1.79 ± 3.29 | 3.11 ± 2.31 | 1.50 ± 0.84 | |
| UO < 0.5 mL/kg/h (SOFA-2 ≥3) | 94/943 (10.0%) | 6/160 (3.8%) | 9/111 (8.1%) | |
| UO < 0.3 mL/kg/h (SOFA-2 = 4) | 57/943 (6.0%) | 3/160 (1.9%) | 7/111 (6.3%) | |
| RRT use | 10 (0.86%) | 13 (0.95%) | 3 (2.46%) | |
| SOFA-1 renal score | 1.34 ± 1.43 | 2.01 ± 1.51 | 0.75 ± 1.21 | |
| SOFA-2 renal score | 0.72 ± 1.13 | 1.02 ± 1.29 | 0.75 ± 1.18 | |
| Renal score difference | −0.61 ± 1.14 | −0.96 ± 1.52 | −0.01 ± 0.61 | |
| **Cardiovascular System** |  |  |  | |
| Mean arterial pressure (mm Hg) | 78.38 ± 11.41 | 79.36 ± 12.14 | 81.94 ± 10.00 | |
| MAP < 70 mm Hg (SOFA ≥1) | 283/1150 (24.6%) | 314/1355 (23.2%) | 10/119 (8.4%) | |
| Lactate (mmol/L) | 1.82 ± 1.32 | 2.01 ± 1.64 | 2.15 ± 2.09 | |
| Lactate > 2 (SOFA-2 ≥2) | 169/655 (25.8%) | 132/405 (32.6%) | 24/77 (31.2%) | |
| Lactate > 4 (SOFA-2 ≥3) | 30/655 (4.6%) | 33/405 (8.1%) | 10/77 (13.0%) | |
|  | | | |  |
| Lactate > 8 (SOFA-2 = 4) | 5/655 (0.8%) | 7/405 (1.7%) | 3/77 (3.9%) | |
|  |  |  |  | |
| Dopamine use | 29 (2.50%) | 22 (1.62%) | 16 (13.11%) | |
| Dopamine > 5 μg/kg/min (SOFA ≥3) | 18/29 (62.1%) | 12/24 (50.0%) | 7/16 (43.8%) | |
| Dopamine > 15 μg/kg/min (SOFA = 4) | 0/29 (0.0%) | 2/24 (8.3%) | 0/16 (0.0%) | |
| Norepinephrine use | 240 (20.65%) | 298 (21.88%) | 68 (55.74%) | |
| Norepinephrine > 0.1 μg/kg/min | 76/240 (31.7%) | 79/300 (26.3%) | 31/68 (45.6%) | |
|  |  |  |  | |
| *(continue)* | | | |  |
| **Characteristic** | **MIMIC-IV (n=1162)** | **eICU-CRD (n=1362)** | **Amsterdam UMC (n=122)** | |
| Epinephrine use | 6 (0.52%) | 1 (0.07%) | 0 (0.00%) | |
| Epinephrine > 0.1 μg/kg/min (SOFA = 4) | 3/6 (50.0%) | 0/2 (0.0%) | N/A | |
| Dobutamine use | 9 (0.77%) | 10 (0.73%) | 6 (4.92%) | |
| Any vasopressor use | 255 (21.94%) | 322 (23.64%) | 71 (58.20%) | |
| SOFA-1 cardiovascular score | 1.33 ± 1.30 | 1.33 ± 1.17 | 2.36 ± 1.53 | |
| SOFA-2 cardiovascular score | 1.18 ± 1.02 | 1.13 ± 0.82 | 1.74 ± 1.14 | |
| Cardiovascular score difference | −0.15 ± 0.66 | −0.20 ± 0.57 | −0.62 ± 0.78 | |
| **Overall SOFA Score** |  |  |  | |
| Total SOFA-1 score | 5.29 ± 2.83 | 5.97 ± 2.65 | 6.11 ± 3.28 | |
| Total SOFA-2 score | 4.93 ± 3.13 | 4.65 ± 2.88 | 5.11 ± 3.46 | |

Abbreviations: Cr, creatinine; ECMO, extracorporeal membrane oxygenation; HFNC, high-flow nasal cannula; MAP, mean arterial pressure; MV, mechanical ventilation; NIV, noninvasive ventilation; P/F, PaO_2_/FiO_2_; RRT, renal replacement therapy; S/F, SpO_2_/FiO_2_; SOFA, Sequential Organ Failure Assessment; UO, urine output.

Note: Continuous variables presented as mean ± SD; categorical variables as n (%). Score difference = SOFA-2 − SOFA-1. SpO_2_/FiO_2_ calculated only when SpO_2_ < 98% (SOFA-2 criterion). Threshold statistics: n/N (x%) indicates n patients meeting criterion among N with available data.

eTable 9. Pooled Cox Model for 28-Day Mortality Adjusted for Age, Sex, and Database

| **Term** | **HR (95% CI)** | **P value** |
| --- | --- | --- |
| **Concordant Positive vs SOFA-2 Only** | 1.509 (1.416–1.609) | < .001 |
| **SOFA-1 Only vs SOFA-2 Only** | 0.962 (0.855-1.083) | .521 |
| **Concordant Negative vs SOFA-2 Only** | 0.749 (0.699-0.803) | < .001 |
| **Concordant Positive vs SOFA-1 Only** | 1.569 (1.410-1.745) | < .001 |
| **Concordant Positive vs Concordant Negative** | 2.014 (1.919-2.115) | < .001 |
| **SOFA-1 Only vs Concordant Negative** | 1.284 (1.148-1.436) | < .001 |

Abbreviations: HR, hazard ratio; CI, confidence interval. Note: A pooled Cox proportional hazards model for 28-day ICU mortality was fitted across all 3 databases and adjusted for age, sex, and database. Pairwise hazard ratios were derived from the same fitted model.

eTable 10. Proportion of Missing Data for SOFA Component Variables

| **Organ System** | **Variable** | **Group** | **MIMIC-IV (N=24,521)** | **eICU-CRD (N=12,209)** | **AmsterdamUMCdb (N=4,720)** |
| --- | --- | --- | --- | --- | --- |
| **Respiratory** | PaO₂ | SOFA-1 & SOFA-2 | 7,328 (29.9%) | 5,290 (43.3%) | 20 (0.4%) |
|  | FiO₂ | SOFA-1 & SOFA-2 | 7,453 (30.4%) | 3,056 (25.0%) | 530 (11.2%) |
|  | SpO₂ | SOFA-2 Only | 43 (0.2%) | 137 (1.1%) | 11 (0.2%) |
| **Coagulation** | Platelet count | SOFA-1 & SOFA-2 | 360 (1.5%) | 6,772 (55.5%) | 104 (2.2%) |
| **Liver** | Bilirubin | SOFA-1 & SOFA-2 | 11,175 (45.6%) | 5,411 (44.3%) | 1,658 (35.1%) |
| **Cardiovascular** | Mean arterial pressure | SOFA-1 & SOFA-2 | 45 (0.2%) | 44 (0.4%) | 22 (0.5%) |
| **CNS** | Glasgow Coma Scale | SOFA-1 & SOFA-2 | 61 (0.2%) | 1,159 (9.5%) | 2,236 (47.4%) |
| **Renal** | Creatinine | SOFA-1 & SOFA-2 | 283 (1.2%) | 432 (3.5%) | 117 (2.5%) |
|  | Urine output (6h) | SOFA-2 Only | 3,324 (13.6%) | 9,087 (74.4%) | 256 (5.4%) |
|  | Urine output (12h) | SOFA-2 Only | 4,782 (19.5%) | 9,257 (75.8%) | 265 (5.6%) |
|  | Urine output (24h) | SOFA-1 & SOFA-2 | 1,523 (6.2%) | 9,120 (74.7%) | 124 (2.6%) |

Missing rates were calculated at the patient level among sepsis-positive patients included in the predictive modeling cohort. A variable was considered missing if no valid value was recorded during the first 24 hours after sepsis diagnosis. Abbreviations: CNS, central nervous system.

eFigure 1. Predictive Modeling Development and Validation Framework


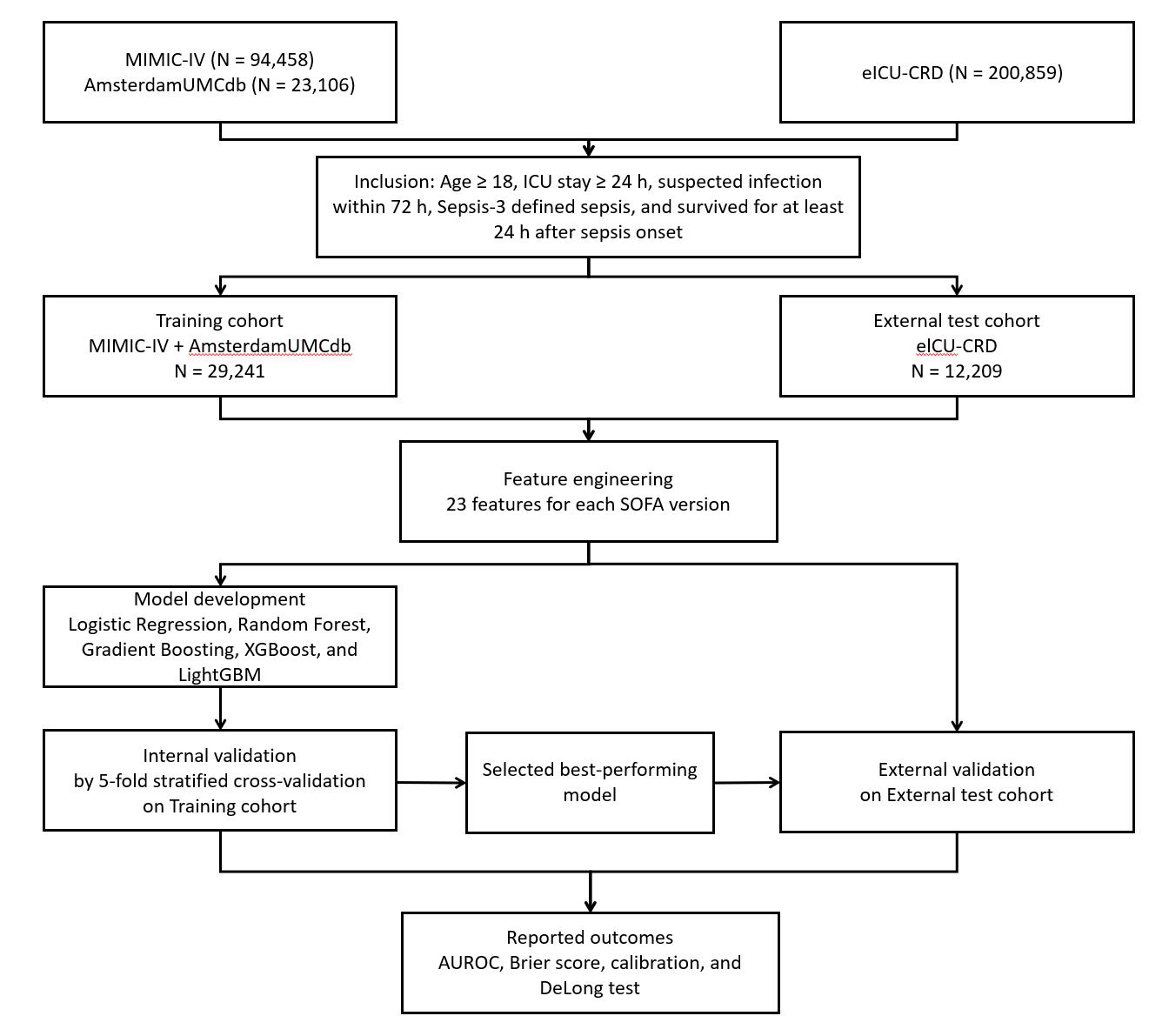


eFigure 2. Diagnostic Concordance and Timeliness Between SOFA-2 and SOFA-1 (MIMIC-IV)


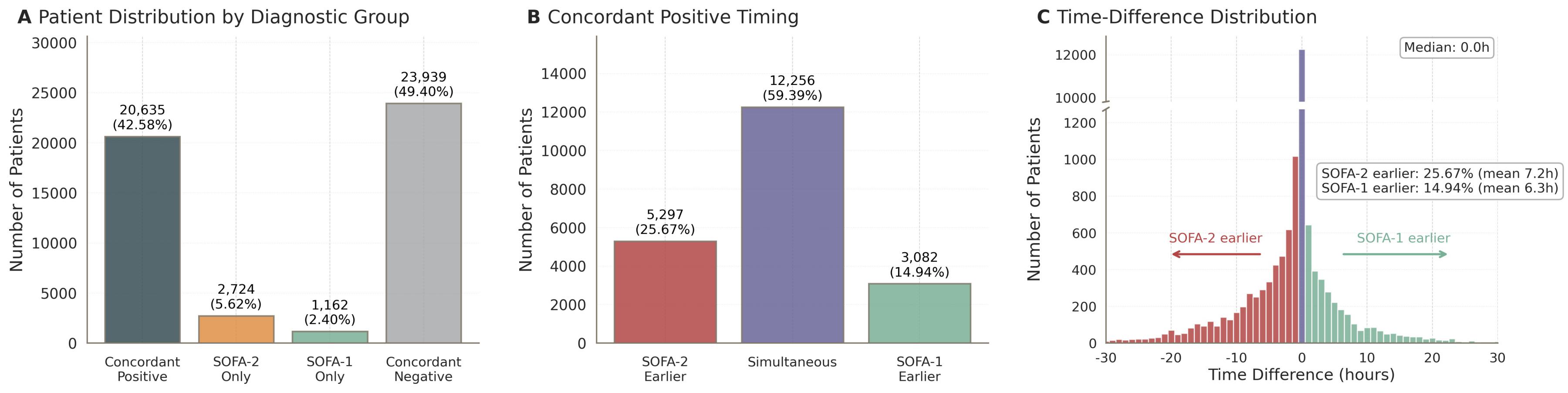


(A) Distribution of diagnostic concordance groups based on SOFA-1 and SOFA-2 sepsis detection. (B) Timing-based subgroups within Concordant Positive patients (SOFA-1 earlier, Simultaneous, or SOFA-2 earlier). (C) Distribution of diagnostic time differences among concordant positive patients, calculated as the SOFA-2 diagnosis time minus the SOFA-1 diagnosis time.

eFigure 3. Diagnostic Concordance and Timeliness Between SOFA-2 and SOFA-1 (eICU-CRD)


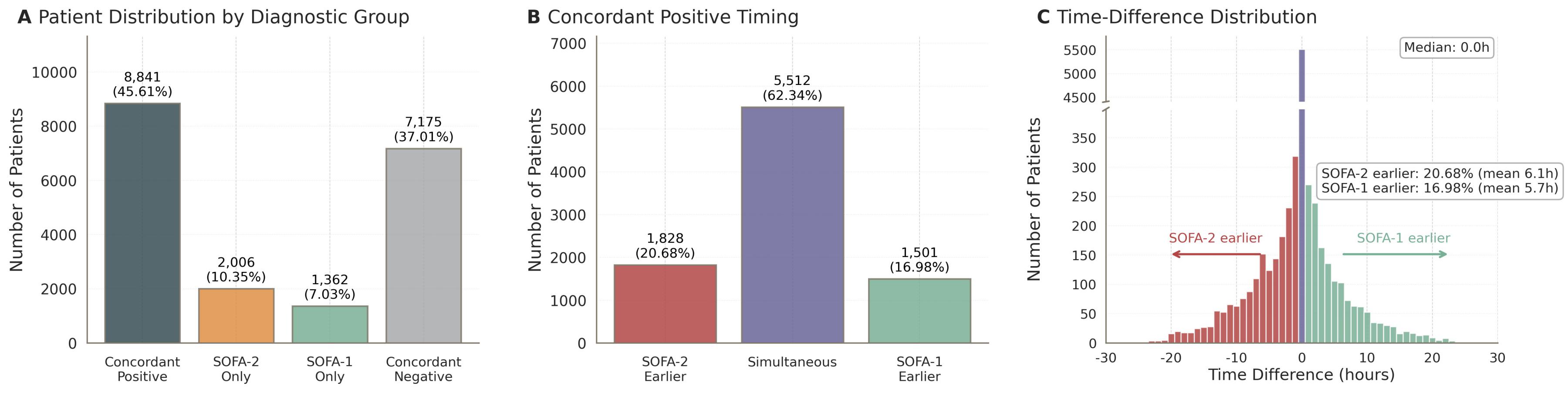


(A) Distribution of diagnostic concordance groups based on SOFA-1 and SOFA-2 sepsis detection. (B) Timing-based subgroups within Concordant Positive patients (SOFA-1 earlier, Simultaneous, or SOFA-2 earlier). (C) Distribution of diagnostic time differences among concordant positive patients, calculated as the SOFA-2 diagnosis time minus the SOFA-1 diagnosis time.

eFigure 4. Diagnostic Concordance and Timeliness Between SOFA-2 and SOFA-1 (AmsterdamUMCdb)


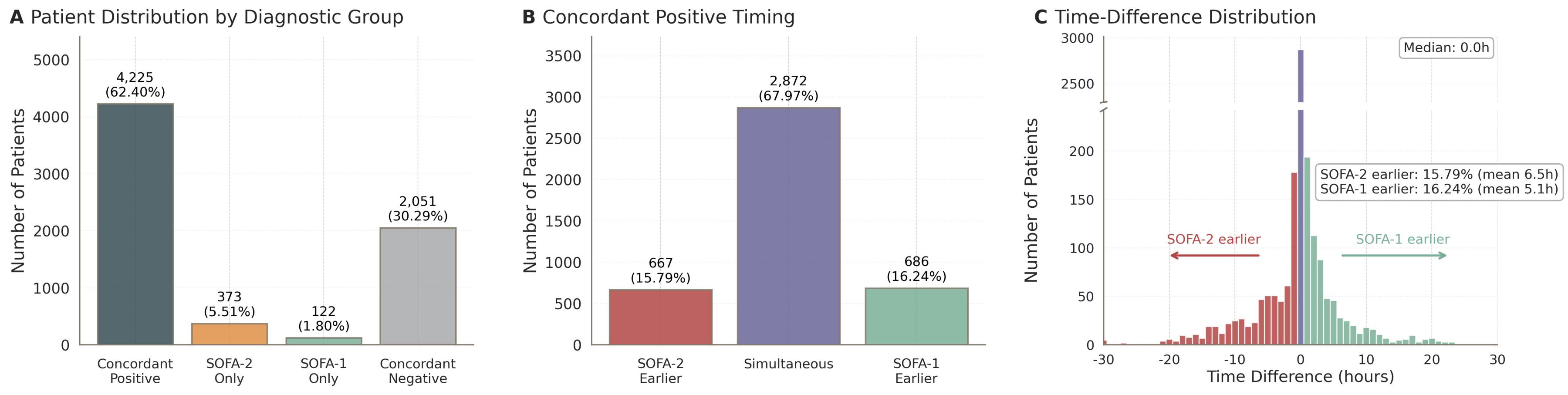


(A) Distribution of diagnostic concordance groups based on SOFA-1 and SOFA-2 sepsis detection. (B) Timing-based subgroups within Concordant Positive patients (SOFA-1 earlier, Simultaneous, or SOFA-2 earlier). (C) Distribution of diagnostic time differences among concordant positive patients, calculated as the SOFA-2 diagnosis time minus the SOFA-1 diagnosis time.

eFigure 5. Clinical Outcomes by Diagnostic Concordance Group (MIMIC-IV)


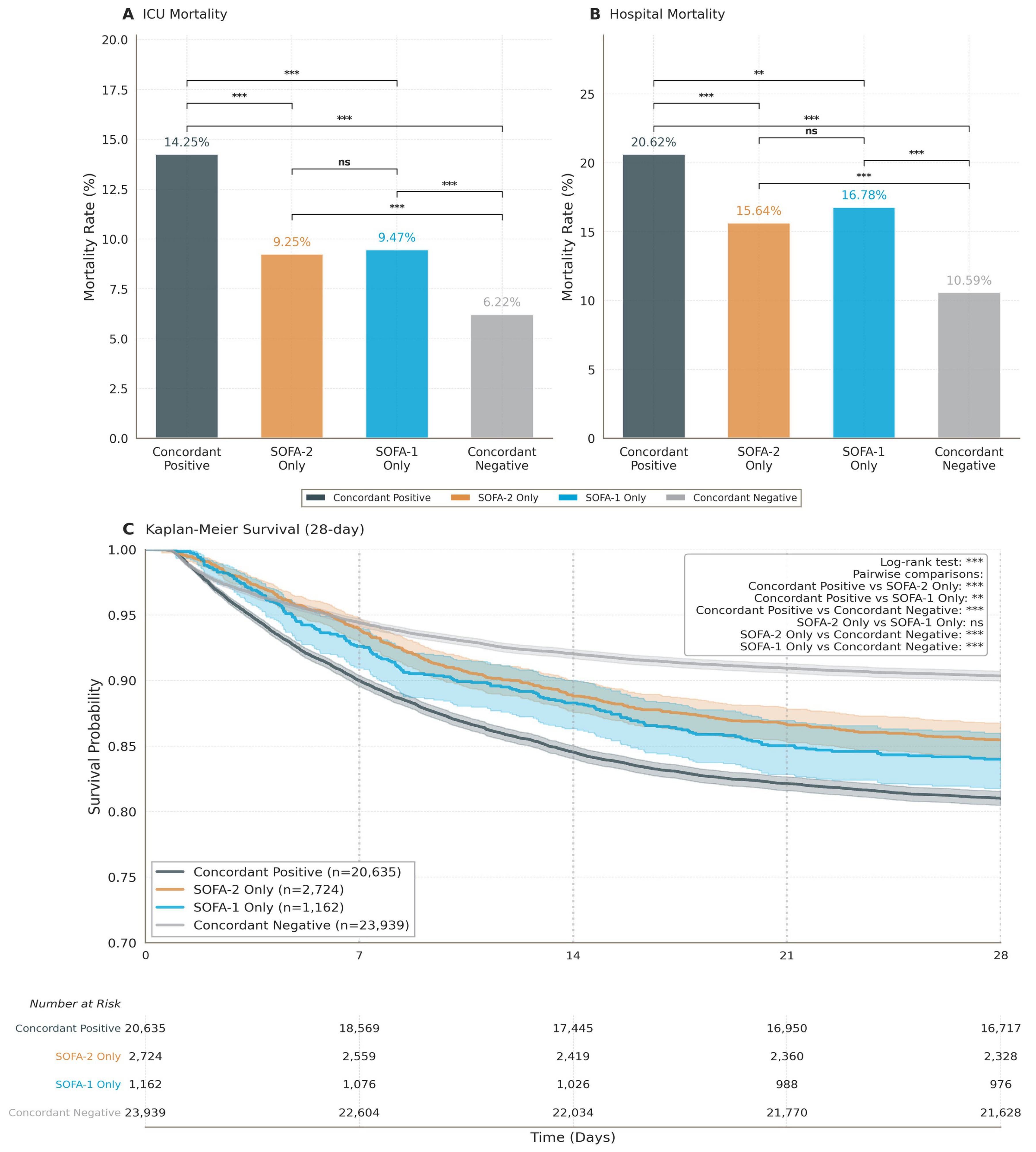


(A) ICU mortality across four groups. (B) Hospital mortality across four groups. (C) Kaplan-Meier survival curves showing 28-day survival by group. ns indicates no significant difference; *** indicates P < 0.001; ** indicates P < 0.01; * indicates P < 0.05.

eFigure 6. Clinical Outcomes by Diagnostic Concordance Group (eICU-CRD)


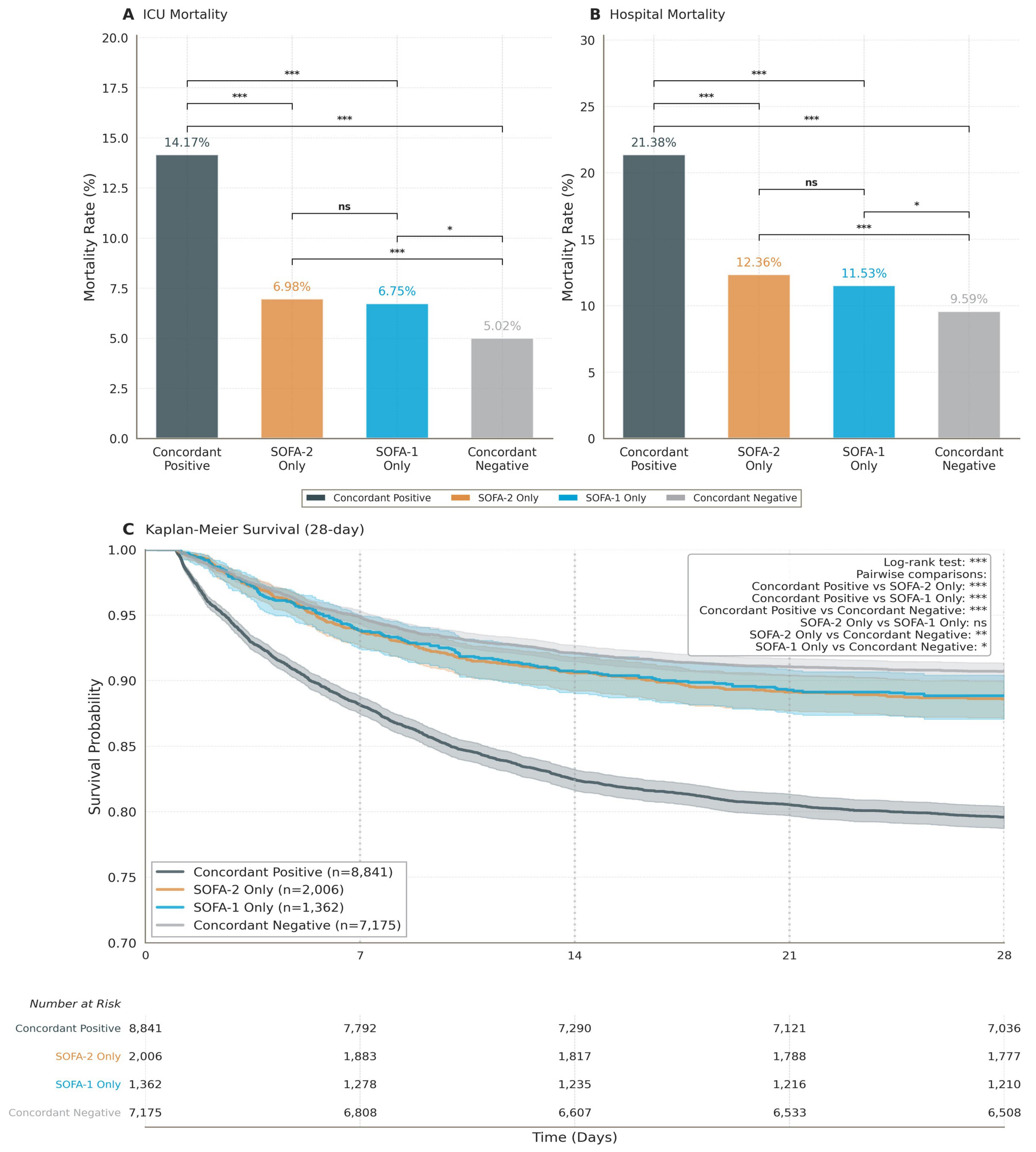


(A) ICU mortality across four groups. (B) Hospital mortality across four groups. (C) Kaplan-Meier survival curves showing 28-day survival by group. ns indicates no significant difference; *** indicates P < 0.001; ** indicates P < 0.01; * indicates P < 0.05.

eFigure 7. Clinical Outcomes by Diagnostic Concordance Group (AmsterdamUMCdb)


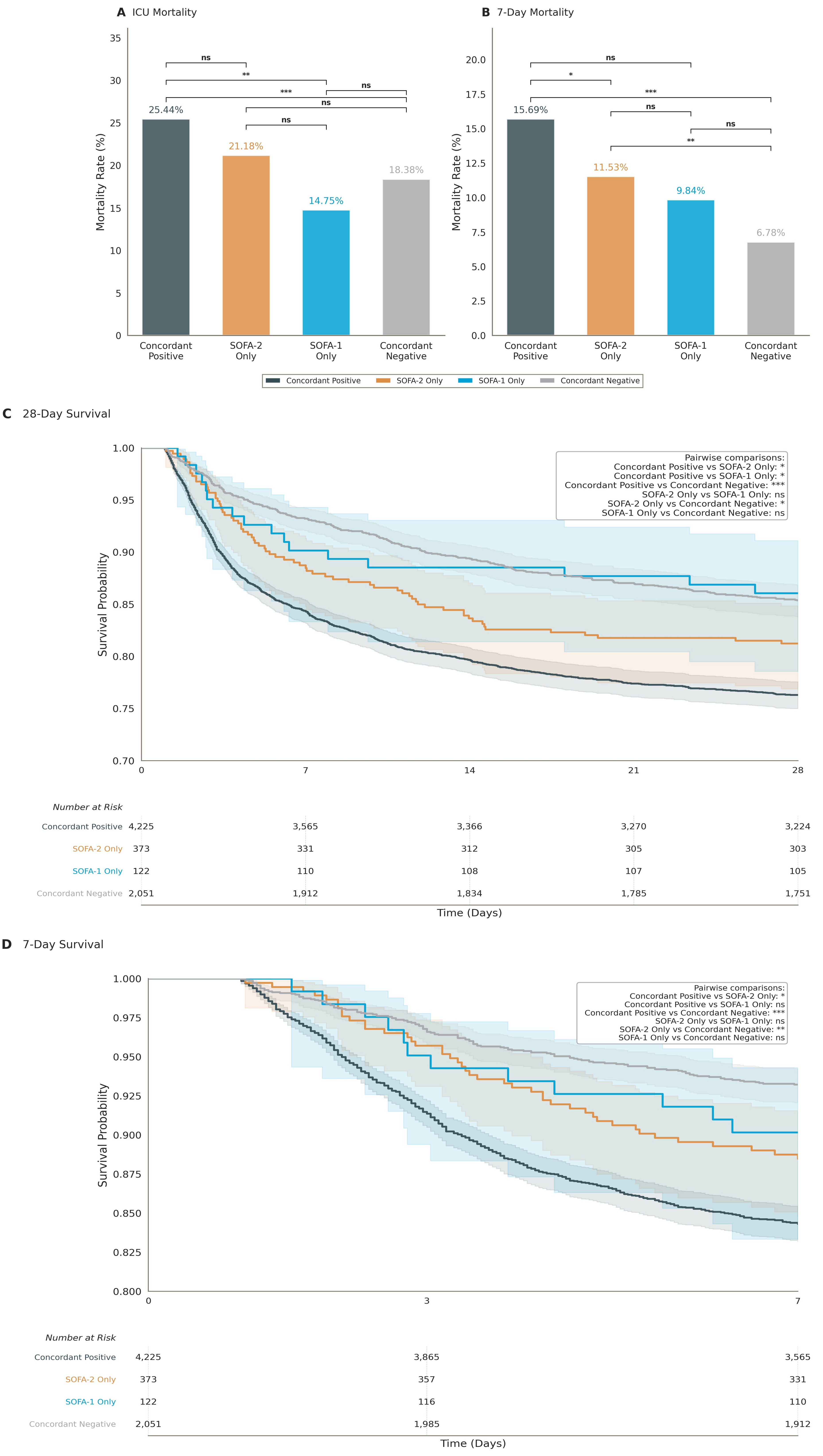


(A) ICU mortality across four groups. (B) Kaplan-Meier survival curves showing 28-day survival by group. ns indicates no significant difference;*** indicates P < 0.001; ** indicates P < 0.01; * indicates P < 0.05.

eFigure 8. Cox Regression Forest Plot (MIMIC-IV)


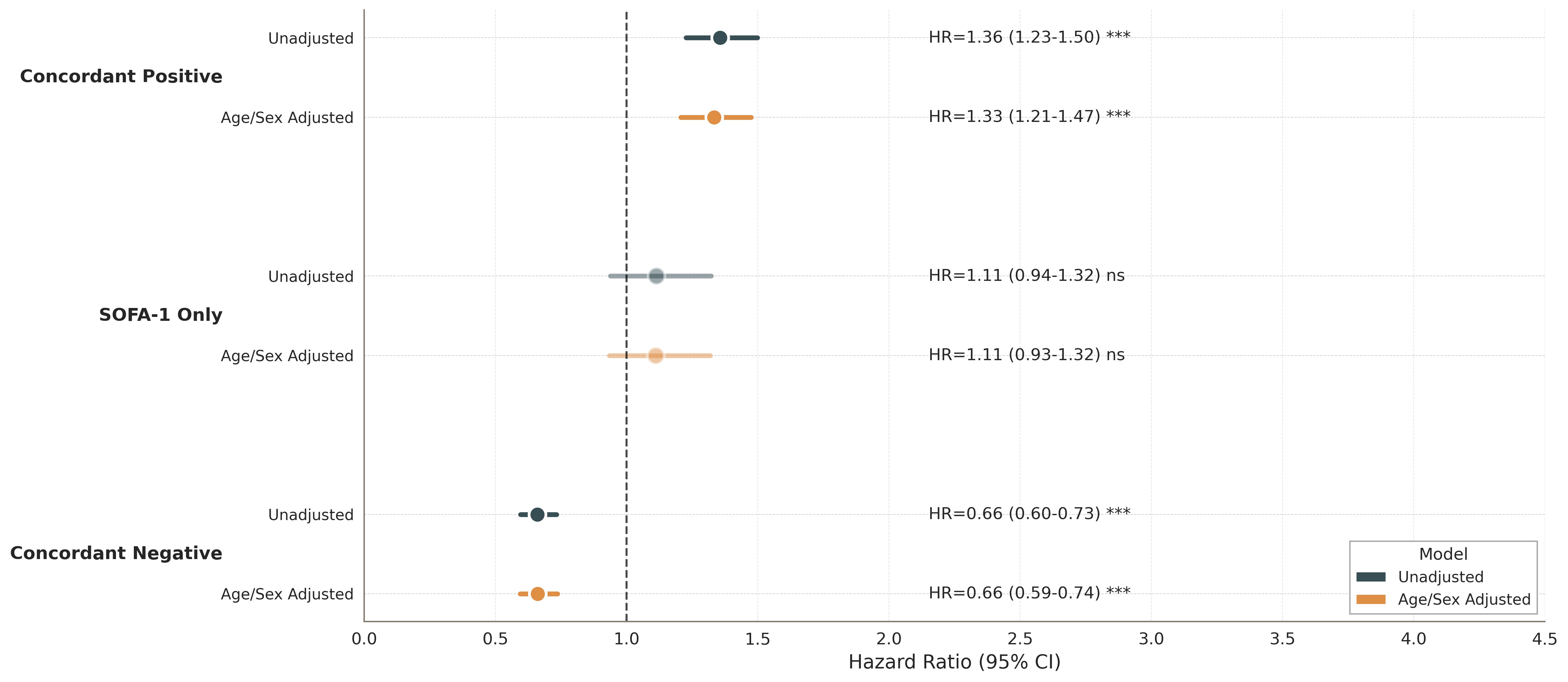


Hazard ratios (HR) for 28-day mortality by concordance group, shown for both unadjusted and age/sex-adjusted models. The reference group is SOFA-2 Only. ns indicates not significant, while *** represents p < 0.001.

eFigure 9. Cox Regression Forest Plot (eICU-CRD)


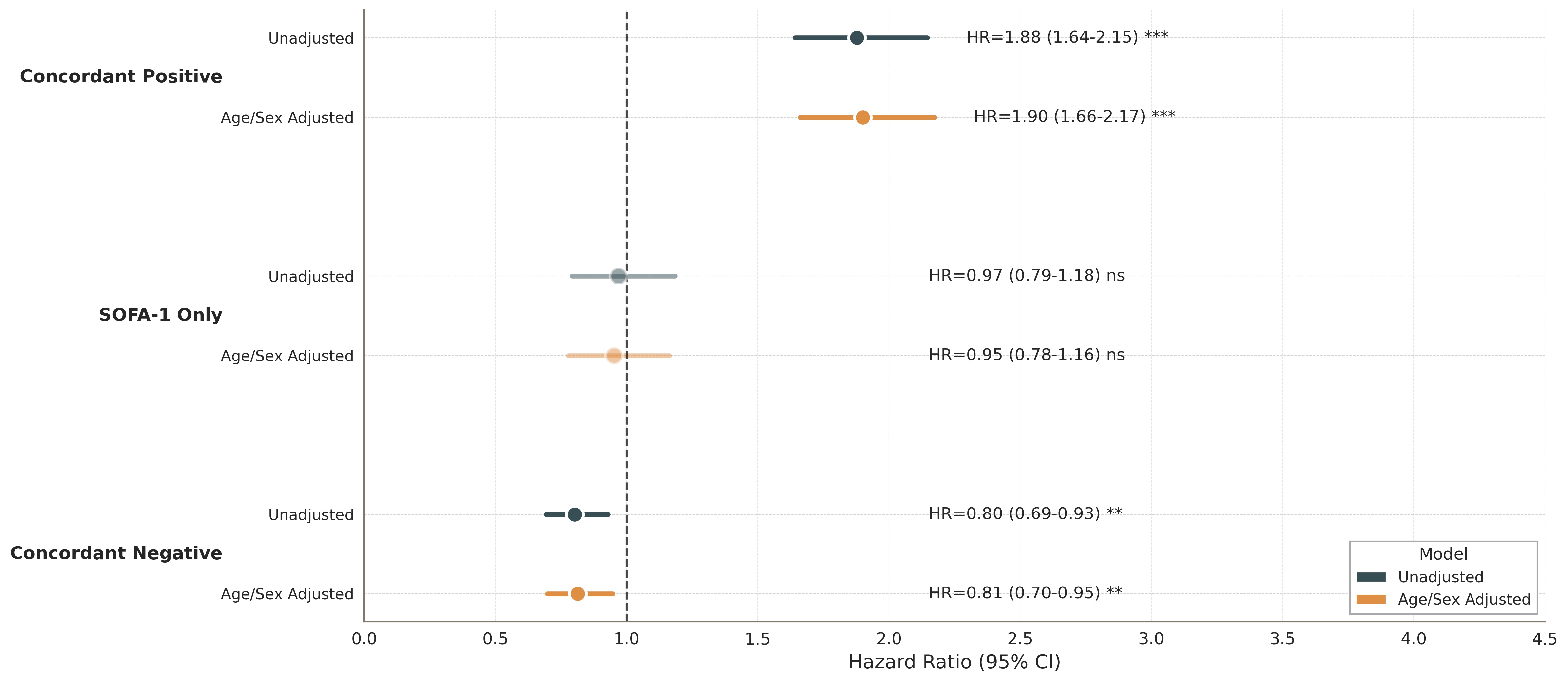


Hazard ratios (HR) for 28-day mortality by concordance group, shown for both unadjusted and age/sex-adjusted models. The reference group is SOFA-2 Only. ns indicates not significant, while *** represents p < 0.001.

eFigure 10. Cox Regression Forest Plot (AmsterdamUMCdb)


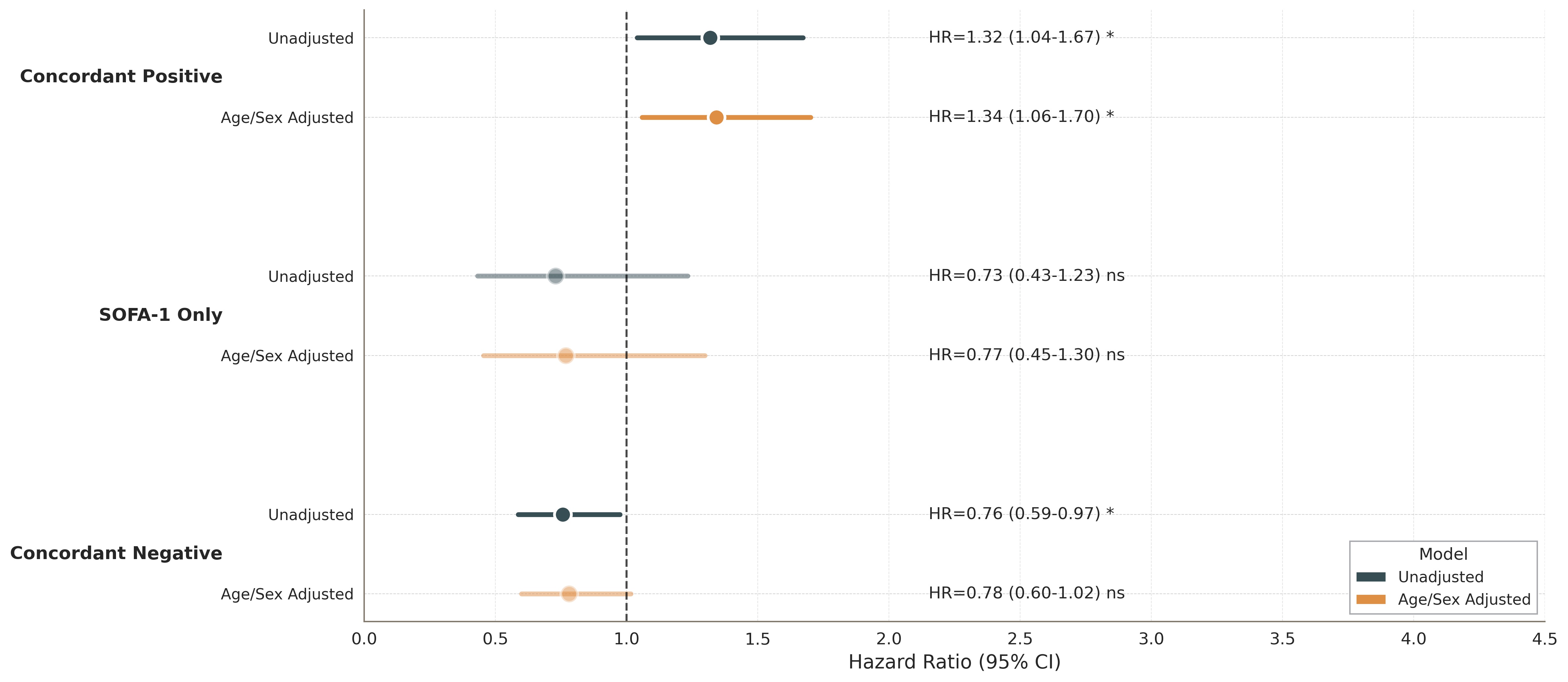


Hazard ratios (HR) for 28-day mortality by concordance group, shown for both unadjusted and age/sex-adjusted models. The reference group is SOFA-2 Only. ns indicates not significant, while *** represents p < 0.001.

eFigure 11. Dumbbell Plot of SOFA-1 vs SOFA-2 Organ Score Differences


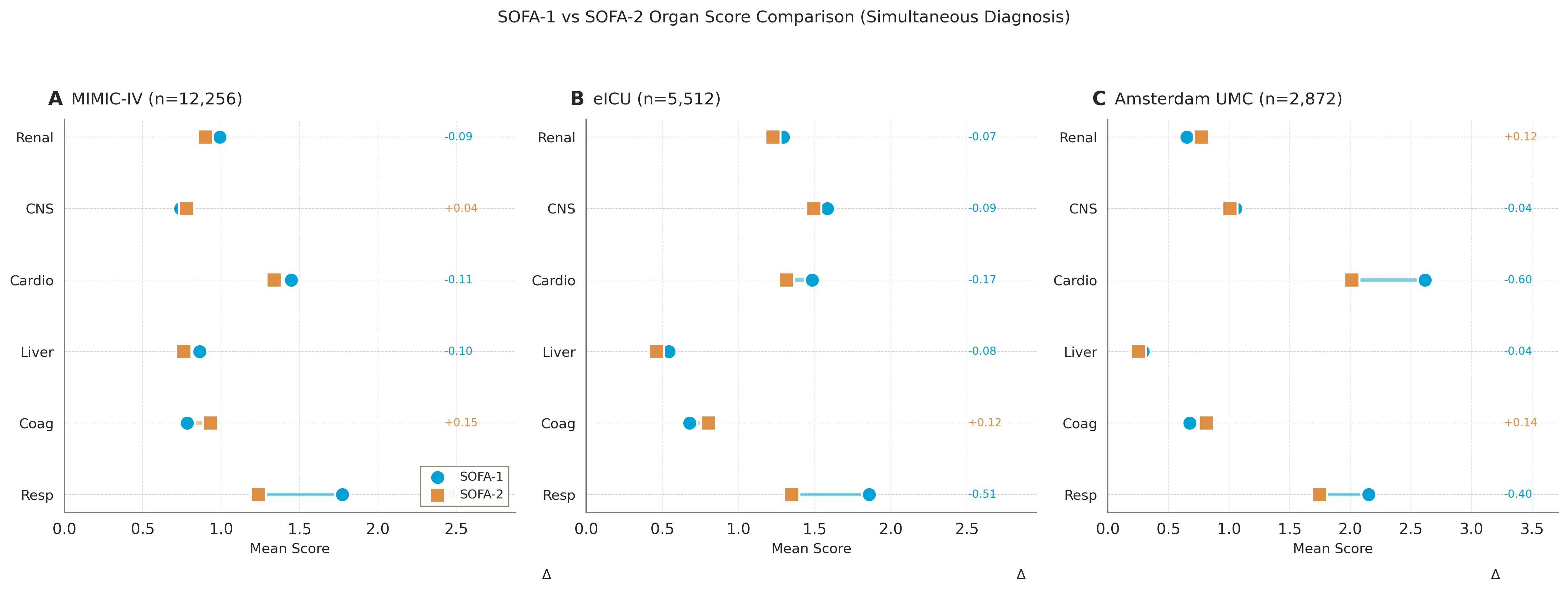


Paired within-database differences in organ subscores at diagnosis among Concordant Positive patients (SOFA-2 minus SOFA-1). Systematic increases are concentrated in respiratory and renal components across all three databases.

eFigure 12. Dynamic SOFA Trajectories (MIMIC-IV)


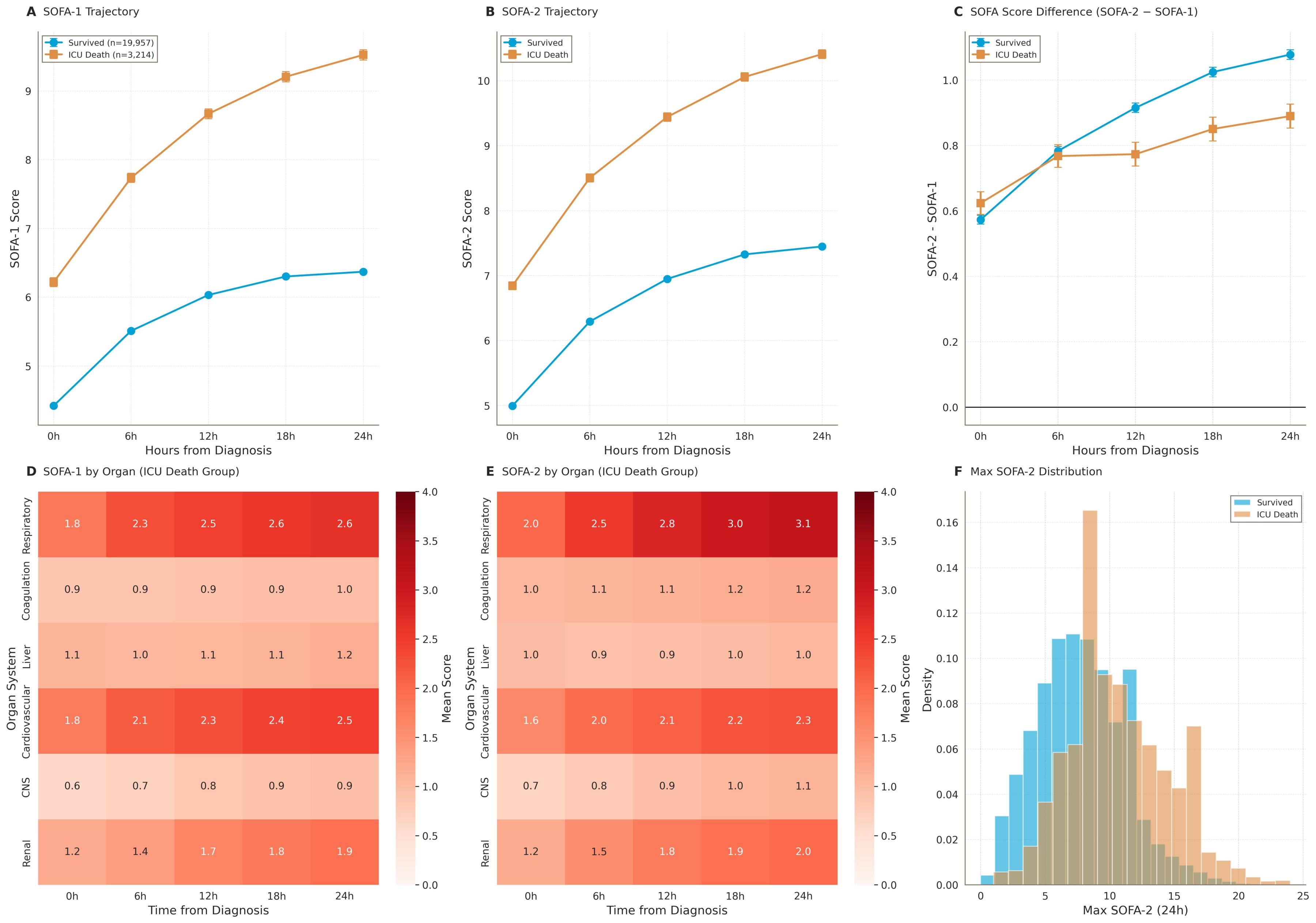


(A) SOFA-1 score trajectories following sepsis detection. (B) SOFA-2 score trajectories following sepsis detection. (C) Difference between SOFA-2 and SOFA-1 scores (SOFA-2 - SOFA-1). (D–E) Organ-specific SOFA heatmaps in patients who died in the ICU. (F) Distribution of maximum SOFA-2 scores.

eFigure 13. Dynamic SOFA Trajectories (eICU-CRD)


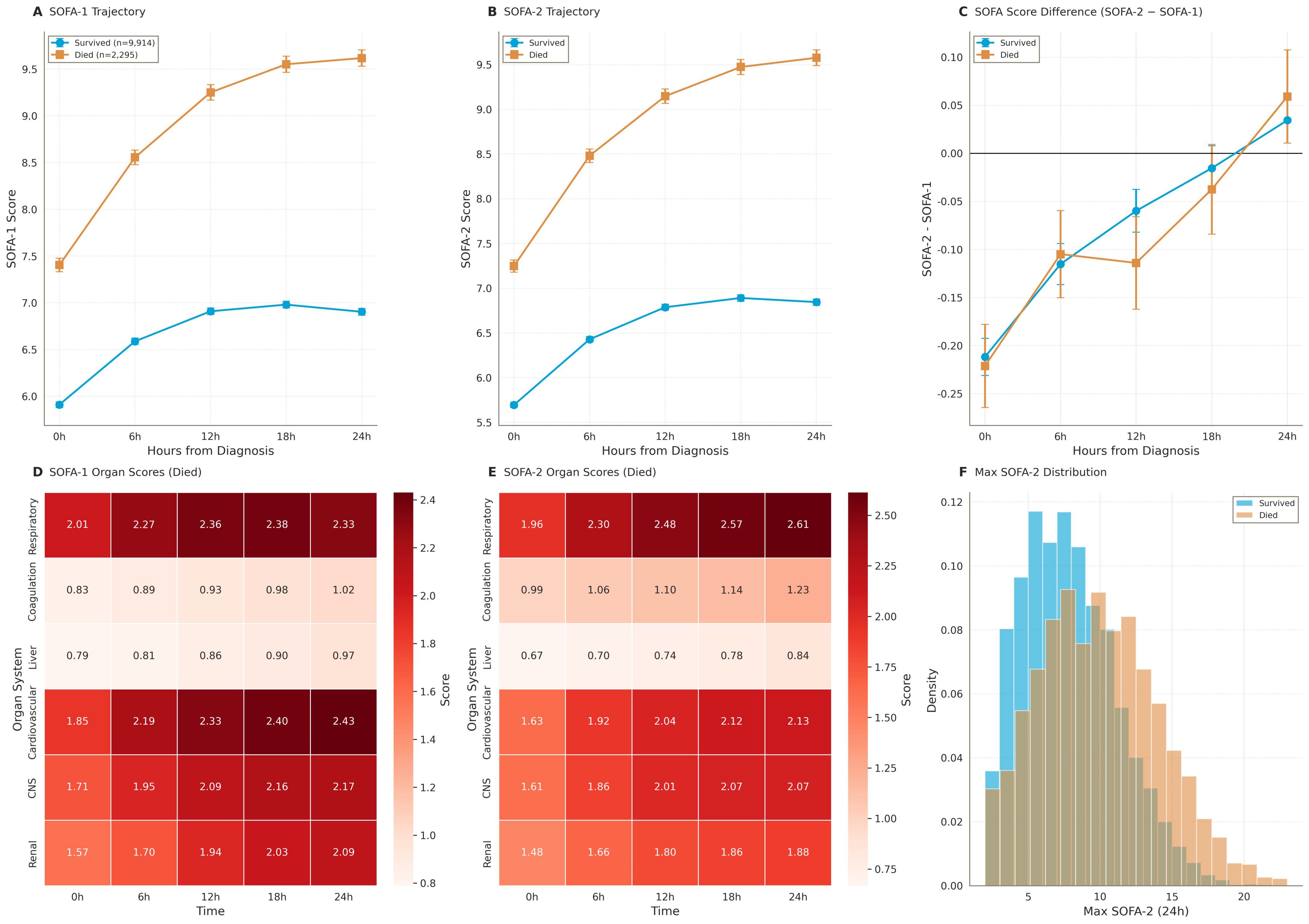


(A) SOFA-1 score trajectories following sepsis detection. (B) SOFA-2 score trajectories following sepsis detection. (C) Difference between SOFA-2 and SOFA-1 scores (SOFA-2 - SOFA-1). (D–E) Organ-specific SOFA component heatmaps in patients who died in the ICU. (F) Distribution of maximum SOFA-2 scores.

eFigure 14. Dynamic SOFA Trajectories (AmsterdamUMCdb)


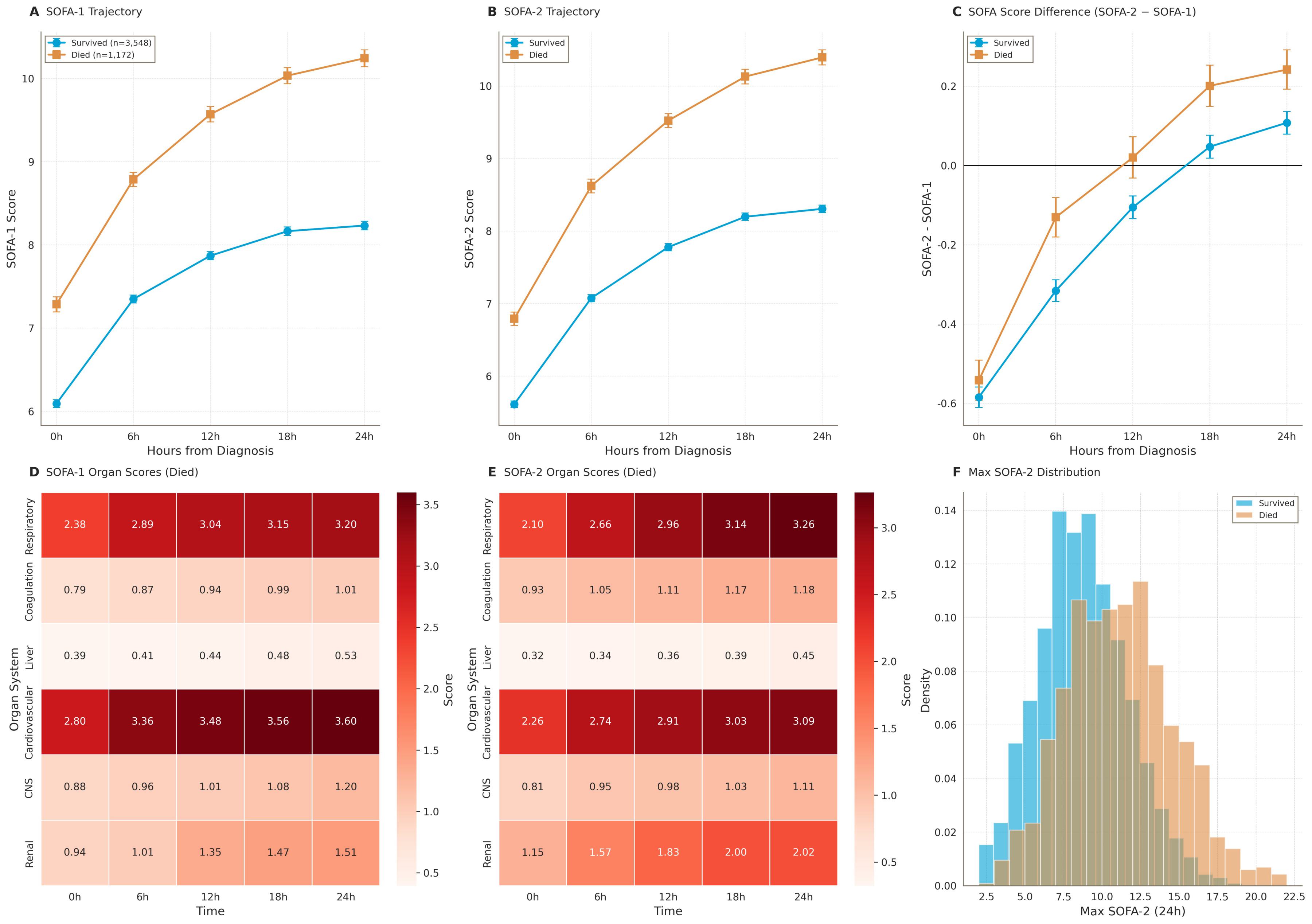


(A) SOFA-1 score trajectories following sepsis detection. (B) SOFA-2 score trajectories following sepsis detection. (C) Difference between SOFA-2 and SOFA-1 scores (SOFA-2 - SOFA-1). (D–E) Organ-specific SOFA heatmaps in patients who died in the ICU. (F) Distribution of maximum SOFA-2 scores.

eFigure 15. Machine Learning Model Comparison (MIMIC-IV)


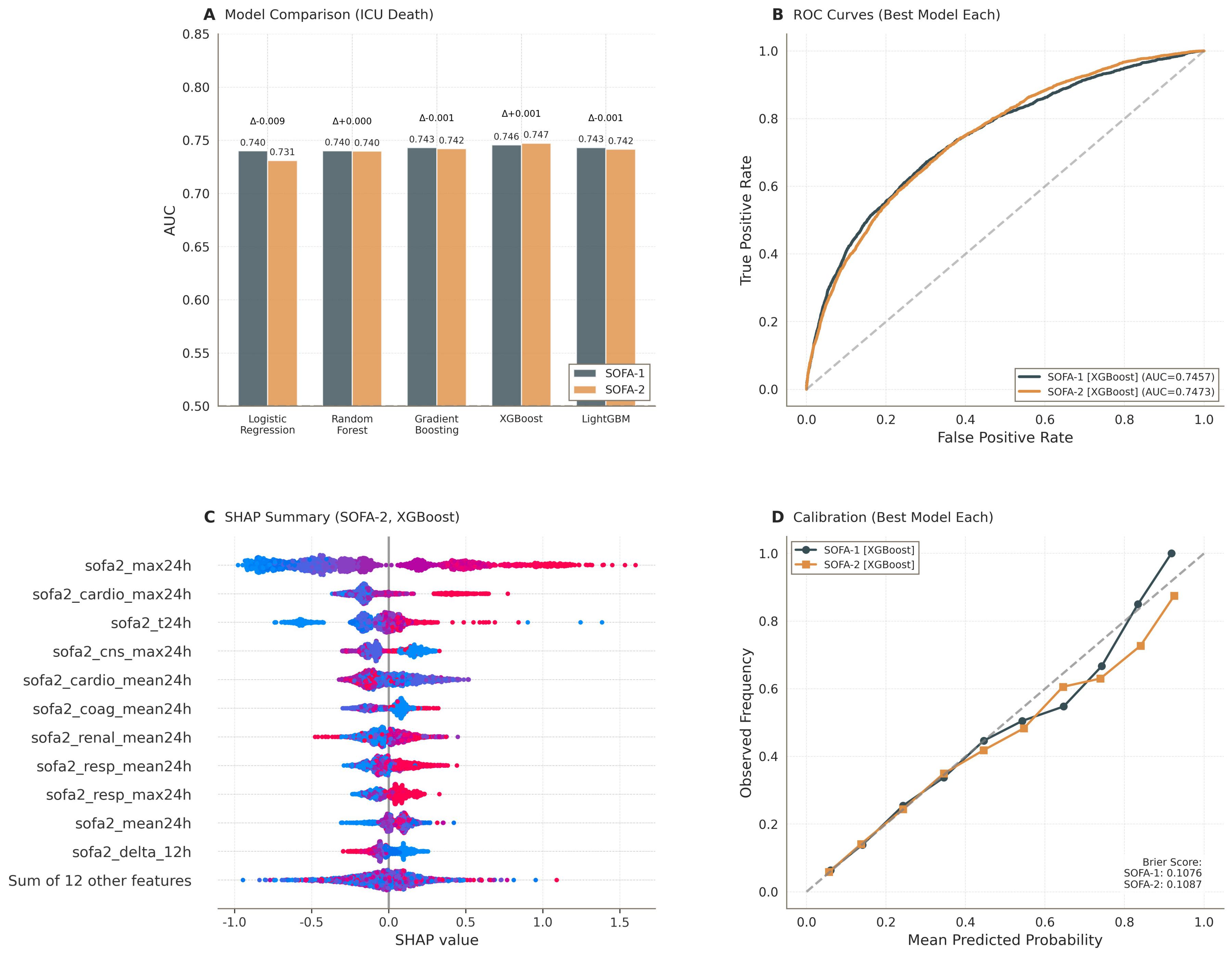


(A) Comparison of AUROC values across five machine learning models using SOFA-1 and SOFA-2 score. (B) ROC curves comparing SOFA-1 and SOFA-2 using the best-performing model. (C) SHAP value summary plot for the best-performing SOFA-2 model in external validation. (D) Calibration curves comparing SOFA-1 and SOFA-2 using the best-performing model in the external validation cohort.

eFigure 16. Machine Learning Model Comparison (eICU-CRD)


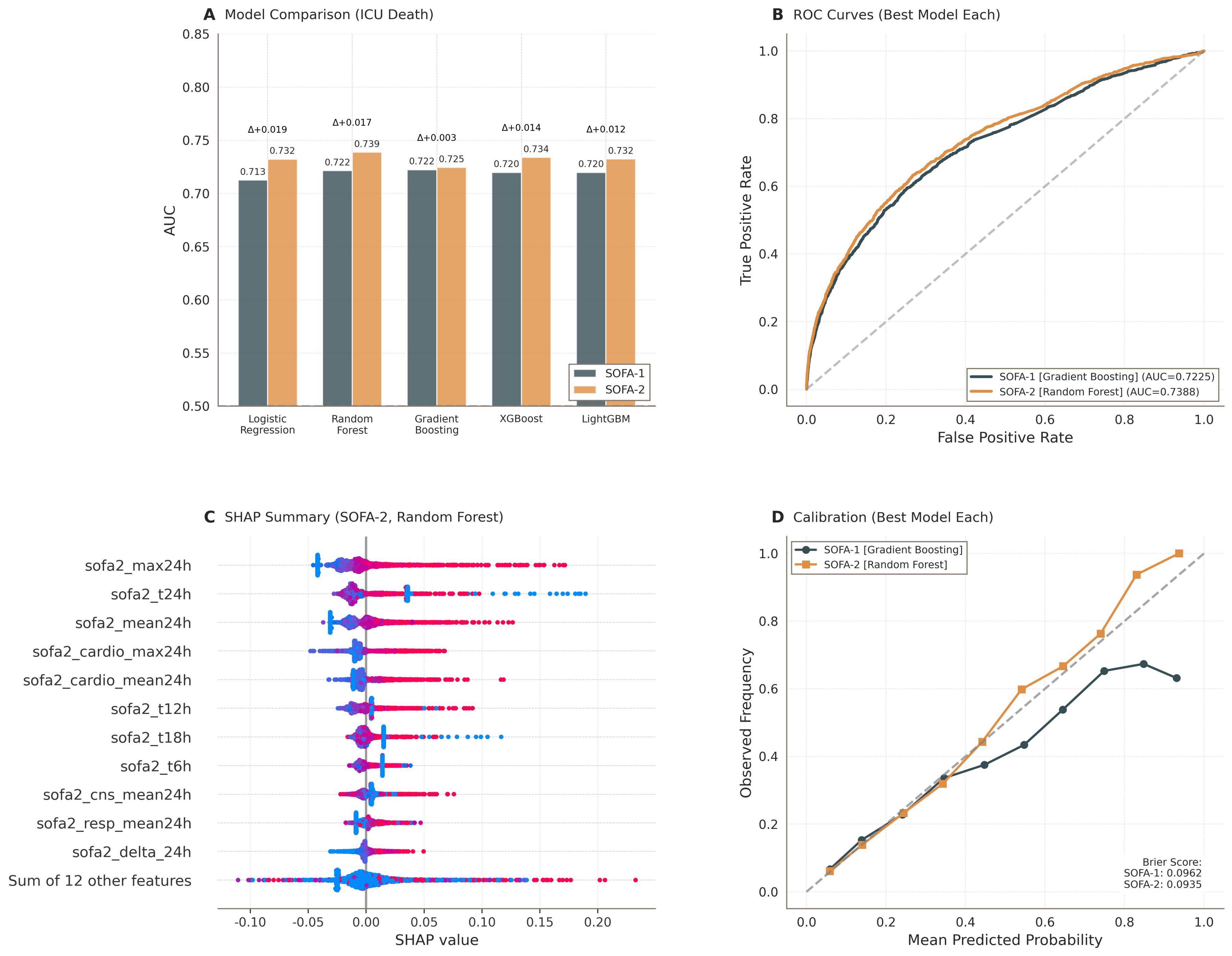


(A) Comparison of AUROC values across five machine learning models using SOFA-1 and SOFA-2 score. (B) ROC curves comparing SOFA-1 and SOFA-2 using the best-performing model. (C) SHAP value summary plot for the best-performing SOFA-2 model in external validation. (D) Calibration curves comparing SOFA-1 and SOFA-2 using the best-performing model in the external validation cohort.

eFigure 17. Machine Learning Model Comparison (AmsterdamUMCdb)


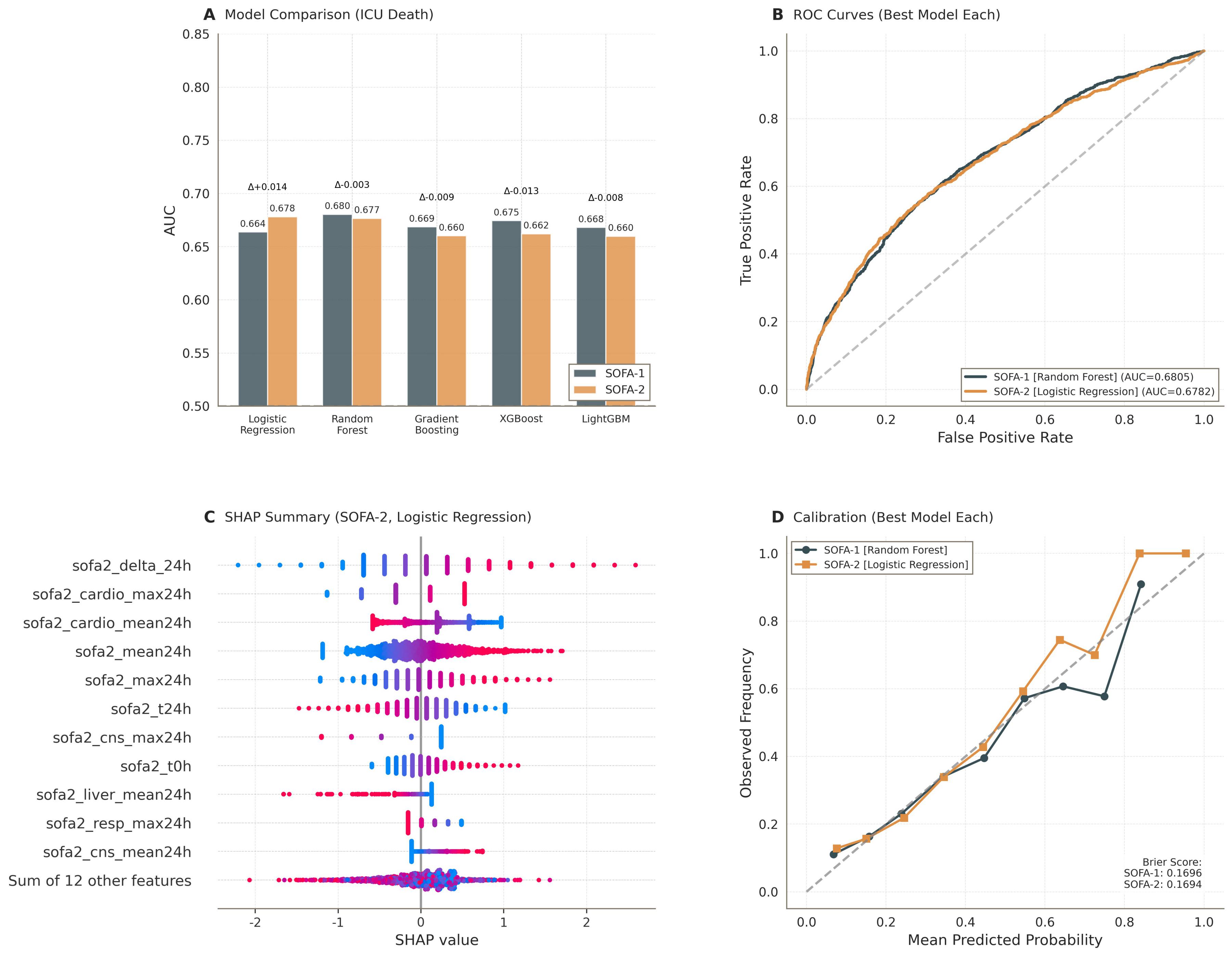


(A) Comparison of AUROC values across five machine learning models using SOFA-1 and SOFA-2 score. (B) ROC curves comparing SOFA-1 and SOFA-2 using the best-performing model. (C) SHAP value summary plot for the best-performing SOFA-2 model in external validation. (D) Calibration curves comparing SOFA-1 and SOFA-2 using the best-performing model in the external validation cohort.

eFigure 18. SHAP Summary Plot for SOFA-1 Features


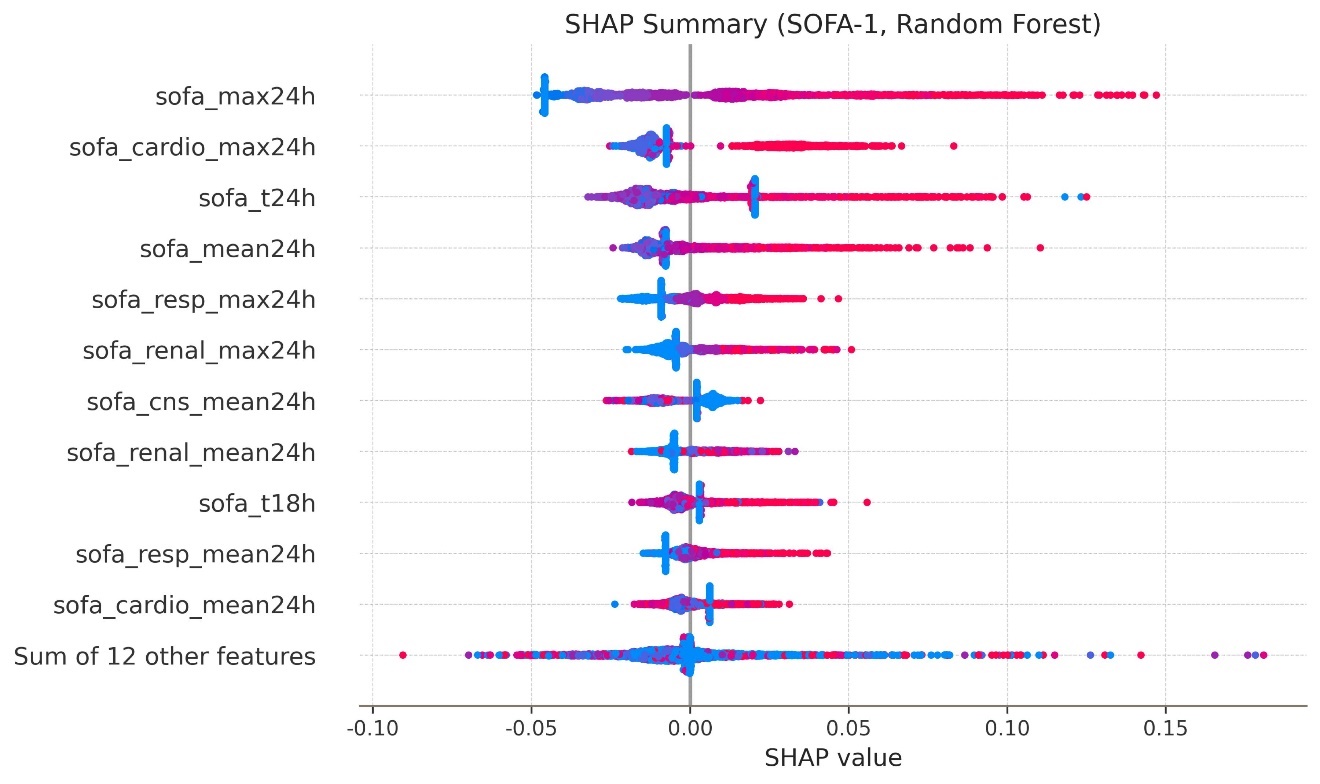


SHAP summary plot for the best-performing SOFA-1 model in external validation.

eFigure 19. Overlap Between Infection and Sepsis Definitions in MIMIC-IV


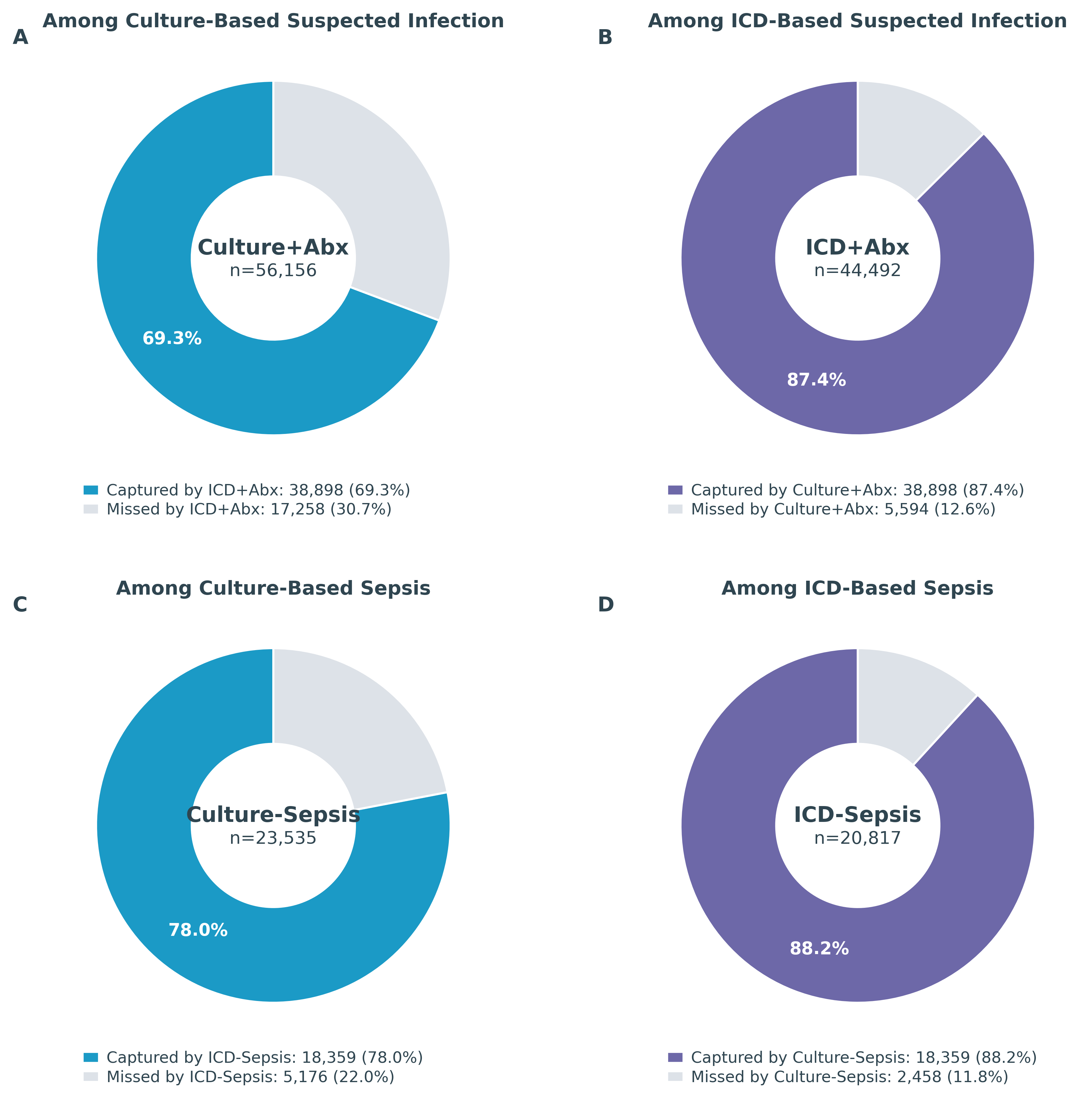


(A) Among patients identified using the culture + antibiotics–based suspected infection definition (n = 56,156), 69.3% also met the ICD + antibiotics definition. (B) Among patients identified using the ICD + antibiotics–based suspected infection definition (n = 44,492), 87.4% also met the culture + antibiotics definition. (C) Among patients with culture-based sepsis (n = 23,535), 78.0% were also classified as septic by the ICD-based definition. (D) Among patients with ICD-based sepsis (n = 20,817), 88.2% were also classified as septic by the culture-based definition.

eFigure 20. Sensitivity Analysis Comparing Mixed-Definition and Culture-Based Suspected Infection Definitions


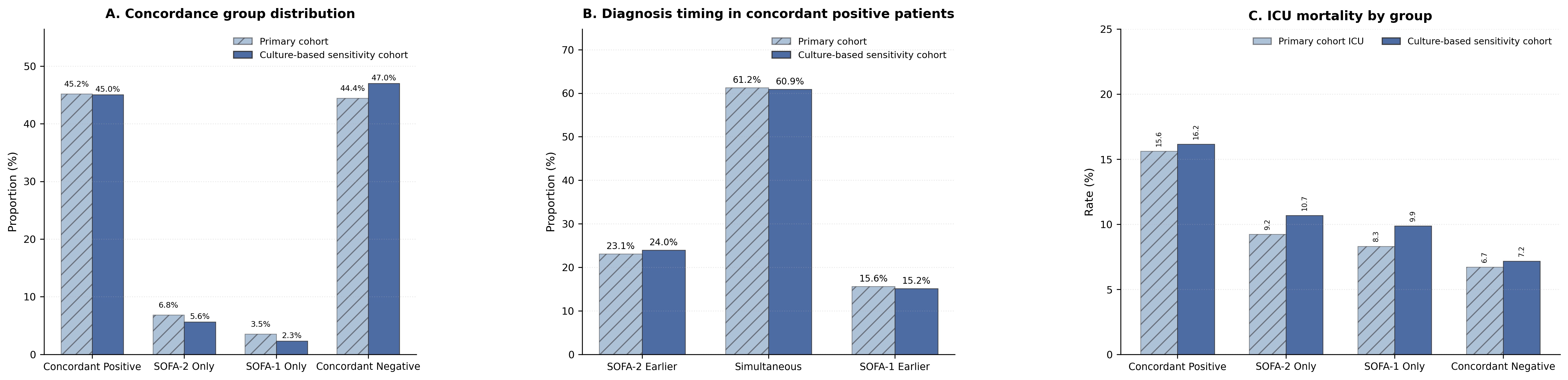


(A) Distribution of diagnostic concordance groups under the mixed-definition and culture-based suspected infection definitions. (B) Timing-based subgroups within Concordant Positive patients. (C) ICU Mortality rates under both definitions.

References

1. Johnson AEW, Bulgarelli L, Shen L, Gayles A, Shammout A, Horng S, et al. MIMIC-IV, a freely accessible electronic health record dataset. Sci Data. 2023;10:1. <https://doi.org/10.1038/s41597-022-01899-x>.

2. Pollard TJ, Johnson AEW, Raffa JD, Celi LA, Mark RG, Badawi O. The eICU Collaborative Research Database, a freely available multi-center database for critical care research. Sci Data. 2018;5:180178. <https://doi.org/10.1038/sdata.2018.178>.

3. Thoral PJ, Peppink JM, Driessen RH, Sijbrands EJG, Kompanje EJO, Kaplan L, et al. Sharing ICU Patient Data Responsibly Under the Society of Critical Care Medicine/European Society of Intensive Care Medicine Joint Data Science Collaboration: The Amsterdam University Medical Centers Database (AmsterdamUMCdb) Example. Crit Care Med. 2021;49:e563–77. <https://doi.org/10.1097/CCM.0000000000004916>.

4. Vincent JL, Moreno R, Takala J, Willatts S, De Mendonça A, Bruining H, et al. The SOFA (Sepsis-related Organ Failure Assessment) score to describe organ dysfunction/failure. On behalf of the Working Group on Sepsis-Related Problems of the European Society of Intensive Care Medicine. Intensive Care Med. 1996;22:707–10. <https://doi.org/10.1007/BF01709751>.

5. Moreno R, Rhodes A, Ranzani O, Salluh JIF, Berger-Estilita J, Coopersmith CM, et al. Rationale and Methodological Approach Underlying the Development of the Sequential Organ Failure Assessment (SOFA)–2 Score: A Consensus Statement. JAMA Netw Open. 2025;8:e2545040. <https://doi.org/10.1001/jamanetworkopen.2025.45040>.

6. Singer M, Deutschman CS, Seymour CW, Shankar-Hari M, Annane D, Bauer M, et al. The Third International Consensus Definitions for Sepsis and Septic Shock (Sepsis-3). JAMA. 2016;315:801–10. <https://doi.org/10.1001/jama.2016.0287>.

7. Pan W, Xu Z, Rajendran S, Wang F. An adaptive federated learning framework for clinical risk prediction with electronic health records from multiple hospitals. Patterns (N Y). 2024;5:100898. <https://doi.org/10.1016/j.patter.2023.100898>.

8. Liu R, Greenstein JL, Fackler JC, Bembea MM, Winslow RL. Spectral clustering of risk score trajectories stratifies sepsis patients by clinical outcome and interventions received. Elife. 2020;9:e58142. <https://doi.org/10.7554/eLife.58142>.

9. Yang M, Zhuang J, Hu W, Li J, Wang Y, Zhang Z, et al. Enhancing Patient Selection in Sepsis Clinical Trials Design Through an AI Enrichment Strategy: Algorithm Development and Validation. J Med Internet Res. 2024;26:e54621. <https://doi.org/10.2196/54621>.

10. Chandra J, Armengol de la Hoz MA, Lee G, Lee A, Thoral P, Elbers P, et al. A novel Vascular Leak Index identifies sepsis patients with a higher risk for in-hospital death and fluid accumulation. Crit Care. 2022;26:103. <https://doi.org/10.1186/s13054-022-03968-4>.

11. Bennett N, Plečko D, Ukor I-F, Meinshausen N, Bühlmann P. ricu: R’s interface to intensive care data. GigaScience. 2022;12:giad041. <https://doi.org/10.1093/gigascience/giad041>.
